# HLA class I and II associations with common enteric pathogens in the first year of life

**DOI:** 10.1016/j.ebiom.2021.103346

**Published:** 2021-04-25

**Authors:** Sayo E. McCowin, G. Brett Moreau, Rashidul Haque, Janelle A. Noble, Shana L. McDevitt, Jeffrey R. Donowitz, Md Masud Alam, Beth D. Kirkpatrick, William A. Petri, Chelsea Marie

**Affiliations:** a Department of Medicine, Infectious Diseases and International Health, University of Virginia School of Medicine, Charlottesville, VA, USA.; b International Centre for Diarrhoeal Diseases and Research, Dhaka, Bangladesh; c Department of Paediatrics, UCSF School of Medicine, San Francisco, CA, USA.; d Innovative Genomics Institute, University of California, Berkeley, CA, USA.; e Children's Hospital of Richmond at Virginia Commonwealth University, Richmond, VA, USA.; f Department of Microbiology and Molecular Genetics, University of Vermont College of Medicine, Burlington, VT, USA.; g Department of Pathology, University of Virginia School of Medicine, Charlottesville, VA, USA.; h Department of Microbiology, Immunology and Cancer Biology, University of Virginia School of Medicine, Charlottesville, VA, USA.

**Keywords:** Children, Infection, HLA, Diarrhoea, Susceptibility, Bangladesh

## Abstract

**Background:**

genetic susceptibility to infection is mediated by numerous host factors, including the highly diverse, classical human leukocyte antigen (HLA) genes, which are critical genetic determinants of immunity. We systematically evaluated the effect of HLA alleles and haplotypes on susceptibility to 12 common enteric infections in children during the first year of life in an urban slum of Dhaka, Bangladesh.

**Methods:**

a birth cohort of 601 Bangladeshi infants was prospectively monitored for diarrhoeal disease. Each diarrhoeal stool sample was analyzed for enteric pathogens by multiplex TaqMan Array Card (TAC). High resolution genotyping of HLA class I (*A* and *B*) and II (*DRB1, DQA1*, and *DQB1*) genes was performed by next-generation sequencing. We compared the frequency of HLA alleles and haplotypes between infected and uninfected children.

**Findings:**

we identified six individual allele associations and one five-locus haplotype association. One allele was associated with protection: A*24:02 – EAEC. Five alleles were associated with increased risk: A*24:17 – typical EPEC, B*15:01 – astrovirus, B*38:02 – astrovirus, B*38:02 – *Cryptosporidium* and DQA1*01:01 – *Cryptosporidium*. A single five-locus haplotype was associated with protection: A*11:01~B*15:02~DRB1*12:02~DQA1*06:01~DQB1*03:01– adenovirus 40/41.

**Interpretation:**

our findings suggest a role for HLA in susceptibility to early enteric infection for five pathogens. Understanding the genetic contribution of HLA in susceptibility has important implications in vaccine design and understanding regional differences in incidence of enteric infection.

**Funding:**

this research was supported by the National Institute of Health (NIH) and the Bill and Melinda Gates Foundation.

**Research in context**

**Evidence before this study**

Diarrhoeal disease accounts for 10% of mortality in children under five years old. Previous work has reported a wide range of pathogens associated with moderate-to-severe diarrhoea in infants and young children living in low-resource settings. Even in the absence of mortality, long-term health deficits are attributed to diarrhoeal disease during early life. The human leukocyte antigen (HLA) system has been implicated as a primary driver of susceptibility and severity of enteric infection. However, the role of HLA variation in early susceptibility to enteric infections warrants further investigation.

**Added value of this study**

We analyzed the relationship between HLA allele variation and infection with 12 enteric pathogens in infants from birth to one year of age. This study is the first HLA association analysis of susceptibility to enteric infection in children followed prospectively from birth. We identified seven novel associations between HLA class I and II alleles and susceptibility to five enteric pathogens.

**Implications of all the available evidence**

This study builds on our previous findings reporting an association between pathogen-specific diarrhoea in early life and decreased long-term growth and cognitive development. These results further support a genetic role for HLA in susceptibility to infection. The data reported here provides insight on potential pathogenic epitopes that lead to a protective or deleterious immune response. These findings have implications for vaccine design and much-needed therapeutics for pediatric diarrhoeal disease.

Alt-text: Unlabelled box

## 1 Introduction

Enteric infection and diarrhoeal disease have an adverse impact on early childhood development in low-and middle-income countries [1]. Diarrhoeal disease is estimated to be responsible for 10% of child mortality in children under five years old [2]. Even in the absence of mortality, negative growth impacts and poor cognitive development have also been attributed to recurrent enteric infection in infants and young children [3,4].

Diarrhoeal disease caused by enteric infection can arise from a broad range of pathogens including viruses, bacteria, and parasites. Recently, the Global Enteric Multicenter Study (GEMS) reported *Shigella* spp., rotavirus, adenovirus, heat-stable toxin enterotoxigenic *Escherichia coli* (ST-ETEC), *Cryptosporidium* spp., and *Campylobacter* spp. as the most common pathogens associated with moderate-to-severe diarrhoea in infants and young children in low-resource settings [5,6]. Several other enteric pathogens including norovirus GII, sapovirus, enteroaggregative *Escherichia coli* (EAEC), and typical enteropathogenic *Escherichia coli* (EPEC) were also significantly associated with moderate-to-severe diarrhoea [5]. In Bangladesh, the major causes of diarrhoea during the first year of life were *Campylobacter jejuni/coli* (*C. jejuni/coli*), rotavirus, astrovirus, *Shigella*/enteroinvasive *Escherichia coli* (EIEC), ST-ETEC, norovirus GII, sapovirus, astrovirus, and *Cryptosporidium* [7].

Genetic association studies are powerful tools for discovery of genetic variants associated with disease and provide insight into prediction, prevention, and treatment of disease [[8], [9], [10]]. The human leukocyte antigen (HLA) complex is the human version of the major histocompatibility complex (MHC), a gene group that is highly associated with infection, immunity, and autoimmunity [11]. HLA classes I and II molecules expressed on the cell surface present pathogen-derived peptides to CD8+ and CD4+ T-cells, respectively. The highly polymorphic nature of HLA genes and the corresponding specificity of their molecules for peptide ligands are fundamental for immune responses to a broad range of infections. Host HLA genotype has been associated with balance between asymptomatic and symptomatic disease progression for a multitude of pathogens [[12], [13], [14], [15], [16]]. Altogether, variation in HLA genes has been extensively associated with differential susceptibility to both infectious and non-infectious diseases.

In this study, we investigated associations between HLA class I and II genes and 12 common enteric infections in a cohort of 601 infants from a longitudinal birth cohort study in Dhaka, Bangladesh [7,17]. Stool specimens were collected from diarrhoeal episodes during the first year of life and the presence of enteric pathogens was detected using the TaqMan Array Card (TAC) system, a highly sensitive molecular diagnostic [18]. The results of the TAC pathogen detection in this cohort have been published previously [7].

## 2 Methods

### 2.1 Study design and population

The PROVIDE study was a prospective clinical trial of polio and rotavirus vaccine interventions, which enrolled a birth cohort of 700 children and their mothers, from an urban slum in Mirpur Dhaka, Bangladesh. Study design including randomization and sample size calculations have been published previously [17] of the 700 children enrolled in the study, 640 children were HLA genotyped. HLA genotyping was performed at the Children's Hospital Oakland Research Institute. The 640 HLA-genotyped infants were all born to Bangladeshi mothers from the northern ward of Mirpur, Dhaka living in slum conditions [17]. The racial composition for the population of Mirpur, Bangladesh is highly homogenous with 98% of the population identifying as Bengali while the remainder is comprised of an indigenous population [18]. A total of 601 HLA-genotyped children who completed the first year of the study were included in these analyses. All 601 children included in this study were vaccinated against tuberculosis, diphtheria, tetanus, whooping cough, hepatitis B, and haemophilus influenzae type *b*, poliovirus, measles, and rubella. Of the 601 children included in these analyses, 301 children were vaccinated against rotavirus.

### 2.2 Enteric pathogen detection

Twice weekly diarrhoea surveillance occurred in the homes by trained field research assistants. Diarrhoea was defined as three or more abnormally loose stools in 24 h according to the mother. To be considered separate diarrhoeal episodes, a 72 h diarrhoea-free period was required. Children with diarrhoea were referred to the study clinic for evaluation and treatment, and a diarrhoeal stool specimen was collected. Stool samples were evaluated for the presence of enteric pathogens by multiplex TAC [19].

### 2.3 Identification of cases and controls

The 12 enteric pathogens with highest incidence, based on the proportion of positive diarrhoeal stool samples in the PROVIDE cohort, were selected for analysis. Presence of infection for each pathogen was identified by PCR detection of nucleic acid in collected diarrhoeal stool samples. Primers used for detection of pathogens have been reported previously [19]. For each pathogen, cases were defined by detection of pathogen nucleic acid by TAC in diarrhoeal stool (0 <CT-value <35). Controls were defined as no detection of the pathogen nucleic acid by TAC in diarrhoeal stool (CT-value≥35) [19]. Identification of cases and controls for each pathogen was performed in R (version 4·0·2) using the tidyverse package (version 1·3·0) [20,21].

### 2.4 HLA genotyping

HLA sequence data were generated on the Roche 454 GS Junior System. Amplicons were generated from genomic DNA using fusion primers consisting of a locus specific primer on the 3´ end, a 10 bp multiplex ID (MID) tag, and either an “A” or “B” 454 specific primer sequence on the 5´ end. The MID tag served as a sample barcode recognized by Conexio ASSIGN™ATF genotyping software (version 1.1.0.35, Conexio Genomics, Freemantle, Western Australia). Amplicons were purified with AMPure beads (Becton Dickinson, Franklin Lakes, NJ), quantified using PicoGreen (Life Technologies, Foster city, CA), diluted, and pooled into libraries. Emulsion PCR, bead enrichment, and sequencing on the GS Junior System were performed using Titanium Series kits (454 Life Sciences) [22,23]. Allele calls were generated from sequence data using customized versions of Conexio Genomics HLA ASSIGN™ATF genotyping software and Sequence COmpilation and REarrangement (SCORE™) software (Graz, Austria) [24]. All HLA genotyping results in this manuscript are reported at the two-field level. The HLA genotyping system used for this work is exon-based. Only the exons that encode the peptide-binding groove on the HLA molecule were sequenced. These include exons 2 and 3 for HLA class I loci and exon 2 for HLA class II loci. Therefore, alleles that are identical in the sequenced exons but differ elsewhere in the gene cannot be distinguished. For example, DQB1*02:01 and DQB1*02:02 are identical for exon 2 but differ in exon 3. In this report, all instances of these alleles are called DQB1*02:01. Given that the number of HLA alleles named to date is greater than 27,000, a complete list of all potential ambiguities is not feasible. Ambiguities in common alleles that may be of note in this report include DQB1*03:01 vs. DQB1*03:19 and DRB1*14:01 vs. DRB1*14:54.

### 2.5 HLA-pathogen association tests

Alleles at the HLA class I loci *HLA-A, HLA-B* and class II loci *HLA-DRB1, HLA-DQA1*, and *HLA-DQB1* of children were analyzed for associations with 12 common enteric pathogens during the first year of life. Association analyses of HLA genotype data and enteric infection was performed using the Bridging ImmunoGenomic Data-Analysis Workflow Gaps (BIGDAWG) software (version 2·3; IDAWG, Oakland, CA, USA) [25] in R (version 1·2·5033). BIGDAWG is an integrated data-analysis pipeline designed for automated association analyses of highly polymorphic genetic data (e.g., HLA genes). BIGDAWG combines rare variants (expected frequency <5 in cases and controls) to a common class to account for sparse cells in tables as described [25,26]. Nine novel HLA alleles were observed; however, they were binned in the analysis due to rare frequency (Supplementary Table 2). Extended haplotype frequencies were mathematically calculated by an EM algorithm using the BIGDAWG package in R. Association tests were performed at the allele and haplotype level. A false-discovery rate (FDR) cut-off of 0·15 was selected to balance the number of HLA alleles deemed significant for further validation while minimizing potential type 1 error from multiple comparisons. Furthermore, when selecting the FDR cut-off, we also considered the biological plausibility of discovering a true genetic role for HLA in host susceptibility to enteric infection. Jvenn, an integrated data-analysis tool for comparing variables with Venn diagrams, was used to compare children with FDR-significant HLA alleles [27].

### 2.6 Socioeconomic status (SES) and exclusive breastfeeding

Surveys were conducted to collect data regarding the child's environment and socioeconomic status (SES). Infants were analyzed for significant differences in several key metrics for SES deemed relevant to enteric pathogen susceptibility. These variables included source of drinking water, water treatment methods, toilet facility, number of household members, household food availability, parental education, number of living children, number of siblings under five years of age, housing quality (floor, wall, roof), and total monthly income/expenditure. Maximum number of days of exclusive breastfeeding was determined for each child enrolled prior to analysis.

### 2.7 Hardy-Weinberg equilibrium (HWE) analysis

In these analyses, we compared 156 alleles across five HLA loci in a cohort of 601 infants during the first year of life. Testing for Hardy-Weinberg equilibrium (HWE) was performed using BIGDAWG (version 2·3) software in R (version 1·2·5033) on cases and controls. There were no deviations from HWE in this cohort study.

### 2.8 Ethics statement

Parents or guardians of those enrolled in this study consented to the collection and use of their data, which was approved by the Research Review Committee and Ethics Review Committee at the International Centre for Diarrhoeal Disease Research (Dhaka, Bangladesh) and by the Institutional Review Boards (IRB# 15377) at the Universities of Virginia and Vermont prior to implementation (Trial registration: ClinicalTrials.gov NCT01375647). HLA genotyping was approved by the Institutional Review Board of the Children's Hospital and Research Centre Oakland.

### 2.9 Statistical analysis

Statistical analyses were done using the GraphPad Prism software package (version 8·4·2; GraphPad Software, La Jolla, CA, USA). The chi-squared test was used for comparison of categorical variables between groups such as exclusive breastfeeding days. The Benjamini-Hochberg procedure was performed to correct for multiple comparisons across alleles with an FDR of 0·15 (15%) [28]. For this procedure, p-values calculated in the BIGDAWG analyses were adjusted by the ratio of total alleles analyzed per locus and p-value rank order (determined by magnitude) of the designated HLA allele at that locus.

### 2.10 Role of funders

The sources of funding for this study had no role in the design of the study, collection of data, analysis of data, interpretation of data, or authorship of the manuscript.

## 3 Results

### 3.1 HLA classes I and II alleles associated with resistance or susceptibility to specific enteric pathogens

**Fig. 1:**
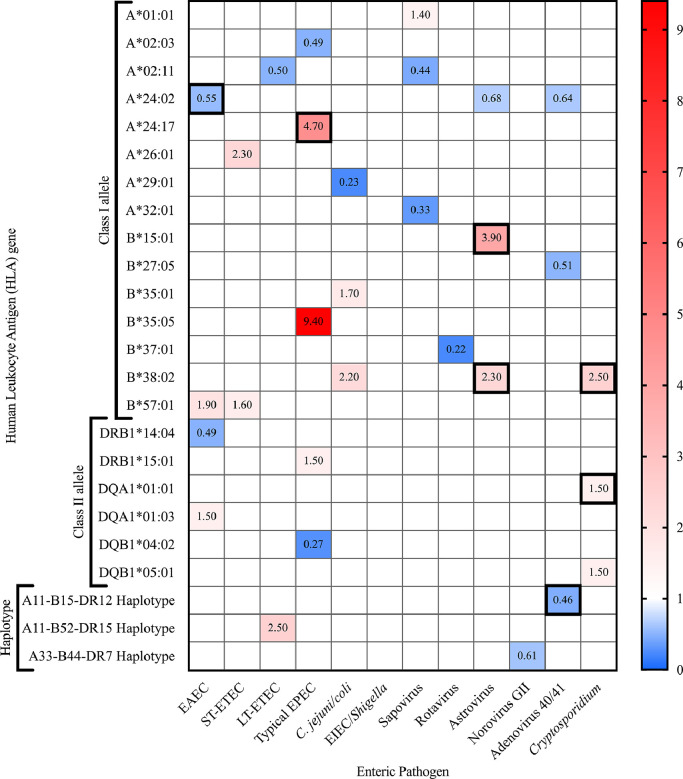
HLA class I and II allele enteric pathogen associations: Associations between five HLA loci (A~B~DRB1~DQA1~DQB1) analyzed and the 12 most common enteric infections in a cohort of 601 infants during the first year of life. Pathogens are arranged by class (bacteria, virus, protozoa). Odds-ratios (OR) for HLA-pathogen associations with *P*-value <0•05 are shown. Red = increased susceptibility to infection, blue = decreased susceptibility to infection. Bolded boxes indicate significant HLA-pathogen associations after correction for multiple comparisons (FDR<0•15). (For interpretation of the references to color in this figure legend, the reader is referred to the web version of this article.)

To investigate genetic susceptibility to enteric infection, we compared incidence of infection for the 12 enteric pathogens with highest incidence (Table 1) in a cohort of 601 infants during the first year of life. For analysis of HLA and susceptibility to rotavirus infection only, we performed the analysis on 300 rotavirus unvaccinated infants. A total of 156 alleles across five loci were compared (33 for *HLA-A*, 58 for *HLA-B*, 39 for *HLA-DRB1*, eight for *HLA-DQA1*, 18 for *HLA-DQB1*) at the two-field level (e.g., A*01:01). We found associations (*P*-value <0·05) for 27 HLA alleles (11 *HLA-A*, ten *HLA-B*, two *HLA-DRB1*, two *HLA-DQA1*, two *HLA-DQB1*) shown in Fig. 1 and listed in Supplementary Table 3. After correction for multiple comparisons, we identified six allele-pathogen associations that withstood an FDR cut-off of 0·15 (Table 2). The distribution of infants with FDR-significant alleles is shown in Supplementary Fig. 1. The six allele-pathogen associations correspond to one viral pathogen, two bacterial pathogens, and one parasite described below.

**Table 1:** Incidence of the 12 most common enteric pathogens in the first year of life.

Pathogen	Controls	Cases	Incidence (%)
EAEC	145	456	76
Adenovirus 40/41	251	350	58
LT-ETEC	261	340	57
ST-ETEC	283	318	53
Typical EPEC	289	312	52
Rotavirus	151	149	50
*C. jejuni/coli*	335	266	44
Norovirus GII	337	264	44
Sapovirus	344	257	43
Astrovirus	394	207	34
EIEC/*Shigella*	458	143	24
*Cryptosporidium*	499	102	17

**Table 2:** HLA class I/II allele-pathogen associations after correction for multiple comparisons**.**

Pathogen	Allele	Controls	Cases	Frequency (controls)	Frequency (cases)	Frequency	*P-*value_Chi-square value	Chi-square value	Degrees of freedom	Odds-Ratio	Confidence Interval Lower 95%	Confidence Interval Upper 95%	*P*-value	*P*-value_adjusted*
EAEC	A*24:02	50	94	0.17	0.10	0.14	0.11	18	12	0.55	0.38	0.82	0.0015	0.018
Typical EPEC	A*24:17	3	15	0.0052	0.024	0.15	0.19	21	16	4.7	1.3	26	0.0072	0.11
Astrovirus	B*15:01	5	10	0.0064	0.024	0.015	0.49	18	19	3.9	1.2	15	0.0082	0.078
	B*38:02	20	23	0.025	0.056	0.040	0.49	18	19	2.3	1.2	4.4	0.0074	0.14
*Cryptosporidium*	B*38:02	29	14	0.029	0.069	0.049	0.069	21	13	2.5	1.2	4.9	0.0056	0.072
	DQA1*01:01	178	51	0.18	0.25	0.21	0.23	9.3	7.0	1.5	1.1	2.2	0.018	0.14

⁎ *P*_adj = Adjusted *p*-value for multiple comparisons by correcting for number of comparisons for each pathogen.

#### 3.1.1 Astrovirus

Astrovirus is a common cause of paediatric diarrhoea and is of particular significance as chronic infections (>14 days) have been linked to infant mortality [29]. In this study, 207 of the 601 infants (34%) had at least one astrovirus-positive diarrhoeal episode (Table 1). We identified two associations for astrovirus infection. The HLA class I allele B*15:01 was associated with increased risk of astrovirus infection (*P*-value= 0·0082 [Chi-square test], OR = 3·9, 95% CI = 1·2–15). This association is the second largest of the six allele-pathogen associations. The HLA class I allele B*38:02 was also associated with increased risk of astrovirus infection (*P*-value= 0·0074 [Chi-square test], OR = 2·3, 95% CI = 1·2–4·4).

#### 3.1.2 Cryptosporidium

*Cryptosporidium* infection has been identified as a leading cause of moderate-to-severe diarrhoea in infants, second only to rotavirus [30]. In fact, cryptosporidiosis was reported to account for 30–50% of deaths in infants and children [31]. In this study, 102 of the 601 infants (17%) had at least one *Cryptosporidium*-positive diarrhoeal episode during the first year of life. We found two associations for *Cryptosporidium* infection. The HLA class I allele B*38:02 was associated with increased risk of *Cryptosporidium* infection (*P*-value = 0·0056 [Chi-square test], OR = 2·5, 95% CI = 1·2–4·9). The B*38:02 allele was also found to be associated with increased risk of astrovirus infection in this cohort. Additionally, the HLA class II allele DQA1*01:01 was associated with increased risk of *Cryptosporidium* infection during the first year of life (*P*-value= 0·018 [Chi-square test], OR = 1·5, 95% CI = 1·1–2·2).

#### 3.1.3 Enteroaggregative *E. coli* (EAEC)

Enteroaggregative *E. coli* (EAEC) was the most common enteric pathogen in this study with 456 of the 601 infants (76%) having at least one EAEC-positive diarrhoeal episode during the first year of life. Infants were also prone to repeat EAEC infection as the average infant had at least two EAEC-positive diarrhoeal episodes. We found that the HLA class I allele A*24:02 was associated with protection from EAEC infection (*P*-value= 0·0015 [Chi-square test], OR = 0·55, 95% CI = 0·38–0·82). The association between HLA-A*24:02 and EAEC is the most statistically significant *P*-value after correction for multiple comparisons (*P*_adj = 0·018) for any enteric pathogen analyzed where EAEC also had the highest incidence of infection in this study.

#### 3.1.4 Typical enteropathogenic *E. coli* (EPEC)

Enteropathogenic *E. coli* (EPEC) is a common cause of bacterial gastroenteritis affecting the developing world and the leading cause of diarrhoea-associated mortality in developing countries for infants under 12 months of age as reported in GEMS.5 Typical EPEC is differentiated as expressing the EPEC adherence factor plasmid (pEAF), a key virulence factor that mediates adherence of EPEC to host cells of the intestine [32]. In this study, 312 of the 601 infants (52%) had at least one typical EPEC-positive diarrhoeal episode during the first year of life. We found a single association for typical EPEC infection: the HLA class I allele A*24:17 was associated with an increased risk of typical EPEC infection (*P*-value = 0·0072 [Chi-square test], OR = 4·7, 95% CI =1·3–26). The A*24:17 allele showed the largest association for risk of infection of any of the six allele-pathogen associations identified in this study.

### 3.2 HLA haplotype associations with enteric pathogen infection

To explore HLA haplotype associations with the common enteric pathogens analyzed here, we compared the incidence of infection in 601 infants during the first year of life. In the cohort of 601 infants a total of 630 unique haplotypes for five loci (*A~B~DRB1~DQA1~DQB1*) (Supplemental Table 4) were compared in these analyses at the two-field level. We analyzed associations between eight unique haplotypes carried by >1% of the infants in cases and controls. We found associations (*P*-value <0·05) for three HLA five-locus haplotypes (Fig. 1). We identified one haplotype demonstrating significance after correction for multiple comparisons (*P*_adj <0·15) for adenovirus 40/41 infection (Table 3).

**Table 3:** HLA class I/II haplotype-pathogen associations after correction for multiple comparisons.

Pathogen	Haplotype (A~B~DRB1~DQA1~DQB1)	Controls	Cases	Frequency (controls)	Frequency (cases)	Frequency	*P*-value _Chi-square value	Chi-square value	Degrees of freedom	Odds-Ratio	Confidence Interval Lower 95%	Confidence Interval Upper 95%	*P*-value	*P*-value_adjusted*
Adenovirus 40/41	11:01~15:02~12:02~06:01~03:01	23	15	0.046	0.021	0.034	0.32	7.0	6.0	0.46	0.22	0.92	0.017	0.10

⁎ *P*_adj = Adjusted *p*-value for multiple comparisons by correcting for number of comparisons for each pathogen.

#### 3.2.1 Adenovirus 40/41

Adenovirus types 40/41 are well established as causes of paediatric acute gastroenteritis during the first year of life. Adenovirus 40/41 infection and paediatric gastroenteritis has been linked to socio-economic status disproportionally impacting low-income and middle-income countries [33]. In this study, 350 of the 601 infants (58%) had at least one adenovirus 40/41 positive diarrhoeal episode during the first year of life. We identified one haplotype association for adenovirus 40/41 infection in our cohort. The A11-B15-DR12 haplotype (HLA-A*11:01~B*15:02~DRB1*12:02~DQA1*06:01~DQB1*03:01) was associated with protection from adenovirus 40/41 infection (*P*-value = 0·017 [Chi-square test], OR = 0·46, 95% CI = 0·22–0·92).

### 3.3 HLA classes I and II alleles associated with resistance or susceptibility to overall diarrhoea

In this study, 529 of the 601 infants (88%) had at least one diarrhoeal stool sample positive for an enteric pathogen during the first year of life. We identified four HLA alleles associated with overall diarrhoea development deemed significant (*P*-value <0·05) (Supplementary Table 4). Of the four HLA alleles we identified one allele, HLA-DQA1*01:03, demonstrating significance after correction for multiple comparisons (Table 4). Interestingly, we also identified an association between HLA-DQA1*01:03 and increased susceptibility to EAEC infection during the first year of life (*P*-value=0·044 [Chi-square test], OR = 1·5, 95% CI = 1·0–2·2). We acknowledge a limitation of this HLA-overall diarrhoea analysis is the low sample size of the control group due to the high incidence of infection in this cohort, specifically with EAEC (76%). This high incidence of EAEC infection during the first year of life may be confounding our association HLA associations with overall diarrhoeal development.

**Table 4:** HLA class I/II allele associations with overall diarrhoea after correction for multiple comparisons.

Pathogen	Allele	Controls	Cases	Frequency (controls)	Frequency (cases)	Frequency	*P*-value_Chi-square value	Chi-square value	Degrees of freedom	Odds-Ratio	Confidence Interval Lower 95%	Confidence Interval Upper 95%	*P*-value	*P*-value_adjusted*
Overall Diarrhoea	DQA1*01:03	14	190	0.097	0.18	0.14	0.079	13	7.0	2.0	1.1	3.9	0.014	0.11

⁎ *P*_adj = Adjusted *p*-value for multiple comparisons by correcting for number of comparisons for overall diarrhoea.

### 3.4 Enteric pathogen susceptibility not driven by differences in exclusive breastfeeding

Beyond socioeconomic status (SES), early childhood susceptibility to enteric infection has also been linked to the duration of exclusive breastfeeding [34,35]. Human breast milk protection likely arises due to a diminished pathogen exposure and breast milk's anti-microbial, anti-inflammatory, and immunoregulatory components [35]. Furthermore, an early transition away from exclusive breastfeeding in the first month of life in infants was observed in the MAL-ED study [36]. We analyzed infants for an association between exclusive breastfeeding and enteric infection. No significant associations between length of exclusive breastfeeding and susceptibility to enteric infection during the first year of life were observed by chi-squared analysis (data not shown). We therefore concluded that this metric is not responsible for observed differences in susceptibility to enteric infection.

## 4 Discussion

We discovered seven novel HLA associations with five common enteric pathogens in infants. Analysis of enteric infections from birth offers insights into the development of host immunity during infancy, particularly in a region endemic for enteric infection. Prospective monitoring from birth allows for examination of SES and genetic factors on risk of early enteric infection between infected and uninfected infants. Of note, we identified several associations with previously reported associations to other infectious diseases, as well as alleles that increased susceptibility across classes of enteric pathogens. Furthermore, to gain insight into the relationship between HLA and pathogen burden we compared CT-values from primary infection for each HLA-pathogen association. Our analysis revealed no significant differences in mean CT-value when comparing children with a protective/susceptible HLA allele to children without [Student's *T*-test]. This does not decrease our confidence in these associations as there are numerous mechanisms by which HLA may impact susceptibility independently of pathogen burden such as varying antigen recognition or altering adaptive immune responses.

Our findings should be considered in relation to those previously published investigating HLA pathogen associations. In a study investigating COVID-19 positive patients genotyped for HLA loci, HLA-B*15:27 was found to be associated with occurrence of COVID-19 infection (*P*-value = 0·001 [Fisher's exact test], OR = 3·59, 95% CI = 1·7–7·5) [37]. The strength of this increased susceptibility association between HLA-B*15:27 and COVID-19 infection is similar to the associations we have identified in our study. This observed increase in susceptibility to COVID-19 suggests the HLA-B*15:27 molecule has reduced recognition of an immunodominant COVID-19 viral antigen when compared to controls. A separate study discovered an association between HLA-DRB1*11:01 (*P*-value <0·00001 [Chi-square test], OR = 2·0, 95% CI = 1·6–2·6) and HLA-DQB1*03:01 (*P*-value <0·00001 [Chi-square test], OR = 2·4, 95% CI = 1·6–3·4) and viral clearance [38]. Again, this HLA-pathogen analysis yielded associations that parallel the strength of those we identified. For this study however, we do acknowledge that these HLA associations have higher significance than those we discovered which we postulate stems from their combining of multiple studies. An HLA association analysis with human immunodeficiency virus type 1 (HIV-1) susceptibility identified HLA-DRB1*01:01 to be protective (*P*-value = 0·0003 [log rank test], incidence response ratio (IRR)= 0·22, 95% CI = 0·06-0·60) supporting the well-established role of class II CD4 effector cells in response to HIV-1 infection [39]. The strength of this association matches several of our identified protective associations. In a congenital study examining 122 foetuses for toxoplasmosis where HLA class II molecules play a crucial role in immunity, HLA-DQA1*01:03 (*P*-value = 0·0002 [Chi-square test], OR = 3·1) and HLA-DQA1*03:02 (*P*-value = 0·0001 [Chi-square test], OR = 9·6) were found to be associated with increased susceptibility to congenital toxoplasmosis [40]. The magnitude of the HLA-DQA1*03:02 association is similar to our HLA-B*35:05 association with typical EPEC but is far stronger than any other HLA association identified. These HLA-pathogen analyses parallel the findings from our study in both the strength of association and methodology.

While all allele-pathogen associations identified in this study are novel, some of the HLA alleles we identified have been implicated in susceptibility to other infections. HLA-B*15:01 has previously been linked to increased incidence of hepatitis C virus (HCV) infection [41], where we identified an increased risk association between this allele and astrovirus infection. HLA-B*38:02 has been associated with reduced risk of disease severity for both dengue haemorrhagic fever and dengue shock syndrome [42,43], however in this study we identified an association for increased risk of both astrovirus and *Cryptosporidium* infection. The association between HLA-B*27 and arthritis is one of the strongest known relationships between an HLA loci and a disease. Here, we identified a relationship between HLA-B*27:05 and protection from adenovirus 40/41 infection during the first year of life. In a study from the UK, where the frequency of HLA-B*27 is roughly 8%, it was found to be associated with 60–90% of patients with reactive arthritis [44]. In our study, 43 of 601 (7·2%) infants harboured an HLA-B*27 allele. Given the high incidence of infection, particularly with causative agents of reactive arthritis such as *Shigella* and *C. jejuni/coli* [45], paired with the frequency of HLA-B*27 it is plausible these infants may experience increased risk for reactive arthritis later in life.

The majority of the associations identified were with HLA class I alleles. We found HLA allele A*24:02 was protective for risk of EAEC infection. This is not surprising as some EAEC strains have been shown to invade and replicate within intestinal epithelial cells *in vitro*. Therefore, it is plausible intracellular antigens from EAEC can be processed and presented via class I. A class I allele associated with protection from infection is likely acting via presentation of a peptide to the receptor of a CD8+ T-cell generating a protective cell mediated immune response. This difference in peptide presentation which confers increased protection from infection may be a consequence of enhanced binding affinity for the peptide resulting in CD8+ T cell activation [46,47].

We also identified class I associations that increased risk of infection: A*24:17 for typical EPEC, B*15:01 and B*38:02 for astrovirus, and B*38:02 for *Cryptosporidium.* Viral replication requires host cell machinery supporting a role for class I allele presentation. While typical EPEC has been characterized as an extracellular pathogen, intracellular EPEC has been described previously in human infection highlighting a potential class I association [48]. Additionally, as *Cryptosporidium* is known to invade intestinal epithelial cells and exist in an intracellular extra-cytoplasmic niche, observation of both classes I and II associations is not surprising [49]. This increased risk of infection association we observe in this study for these class I alleles may be due to a poorer presentation of peptides derived from these pathogens.

HLA class II allele DQA1*01:01 increased risk of *Cryptosporidium* infection. Presentation of bacterial antigens by HLA class II molecules following macroautophagy and lysosomal degradation is critical for the CD4+ T-cell enhanced antibody response to that pathogen [50,51]. We hypothesize that the association between DQA1*01:01 molecule and *Cryptosporidium* infection could be due to an inability to present *Cryptosporidium*-derived antigens.

These results should also be considered in light of some potential limitations. First, to detect enteric infection, we collected diarrhoeal stool samples from infants, which may underestimate cases as sub-clinical infection is common [52,53]. Any infection in an infant that did not result in a corresponding diarrhoeal episode would have gone undetected in our analysis. Second, detection of multiple enteric pathogens during an episode of diarrhoea is common in this population [53]. In these instances, the presence of an enteric pathogen was considered as an infection. Third, though we attempt to correct for multiple comparisons by using the Benjamini-Hochberg procedure, the magnitude of comparisons performed in this analysis may limit findings. Validation of the allele-pathogen associations identified in this study in additional studies could provide valuable insight. Lastly, pathogens with multiple strains were defined based on known shared biological processes and pathogenesis (EAEC, ST-ETEC, typical EPEC, rotavirus, *Cryptosporidium*).

In this analysis, HLA-A*24:02 was associated with protection from EAEC infection during the first year of life. It is notable that we previously identified a protective association between HLA-A*24:02 and *Cryptosporidium* infection in Bangladeshi children ages 2–5 [16]. The identification of multiple enteric pathogen associations for HLA-A*24:02 suggests the peptide binding region for the A*24:02 molecule has a wide repertoire capable of recognizing and presenting peptide ligands derived from EAEC and *Cryptosporidium*. Moreover, we previously identified associations between alleles HLA-B*15, HLA-DQB1*03:01 and haplotype HLA-DRB1*11:01/HLA-DQB1*03:01 with increased incidence of asymptomatic and symptomatic *Cryptosporidium* infection [16]. In this current study HLA-B*38:02 and HLA-DQA1*01:01 were associated with increased risk of *Cryptosporidium* infection. These discrepancies in HLA associations identified and *Cryptosporidium* may be explained by the differences in age of the children as specific alleles could influence duration of immune response into early childhood. Furthermore, the present study used the TAC assay for molecular diagnostics which is reported to have an average sensitivity and specify of 85 and 77% when compared to conventional tests such as the ELISA used by the previous study [19]. In the current study, 102 children (17%) had a *Cryptosporidium*-positive diarrhoeal episode compared to the previous cohort of 58 children (26%). Finally, the previous study examined the association between HLA and *Cryptosporidium* infection, including both symptomatic and asymptomatic infection, while this study focuses on the relationship between HLA and symptomatic *Cryptosporidium* infection (i.e., diarrhoea).

A major strength of this study was the ability to compare HLA genotypes and susceptibility to multiple enteric pathogens during the first year of life, thus permitting the investigation of early immune responses in an immunologically naïve population from an area that has a high incidence of pediatric enteric infection. Second, the children in this study were closely monitored for episodes of diarrhoea in order to collect all relevant samples for pathogen detection. Third, pathogen detection was determined using molecular diagnostics (TAC), allowing for higher sensitivity and quantitative detection of each enteric pathogen when compared to conventional methods [19]. Lastly, this study was performed in a single ethnic population which may have made HLA signals more apparent due to homogeneity of this group.

In summary, our study has successfully identified novel HLA associations with susceptibility to common enteric pathogens in Bangladeshi infants and highlight the impact of host genetics on pathogen susceptibility. Future studies investigating the functional role of HLA-mediated antigen presentation in this setting may be crucial for defining the process by which we develop mucosal immunity to enteric infection and has implications for oral vaccine development.

## 5 Contributors

RH, MA, BDK, and WAP contributed to study design and preparation of ethical protocols. RH and MA enrolled mothers and children for the study. RH, MA, and BDK collected clinical data. JAN and SLM performed HLA genotyping and analysis at Children's Hospital Oakland Research Institute. SEM and GBM performed the statistical data analyses. SEM, WAP, CM, JD, GBM, and JAN contributed to interpretation of data. WAP and CM directed the research. SEM, JAN, WAP, and CM wrote the manuscript. All authors have read and approved the final version of this manuscript.

## Data sharing statement

The data for this study is made available by URL (https://github.com/gbmoreau/HLA_Enteric_Pathogens) to the scientific community for research purposes. The contents of these data includes de-identified individual participant data that underlie the results (text, tables, figures, appendices) reported in this research article. The study protocol has previously been made available by Kirkpatrick and colleagues [17].

## Declaration of Competing Interest

JD reports grants from National Institutes of Health (NIH), Eunice Kennedy Shriver National Institute of Child Health and Human Development, grants from the Bill and Melinda Gates Foundation, grants from Pantheryx, Inc, outside the submitted work. WAP reports grants from NIH and grants from Gates Foundation, during the conduct of the study; other grants from TechLab outside the submitted work. CM reports grants from the NIH and funding from Bill and Melinda Gates Foundation, during the conduct of the study. All other authors have nothing to disclose.

## Appendix Supplementary materials

Image, application 1

Image, application 2
