# Supplementary material for: HLA class I and II associations with common enteric pathogens in the first year of life
Source: eBioMedicine. 2021 Apr 25;67:103346. doi: 10.1016/j.ebiom.2021.103346 (PMC8093888; doi:10.1016/j.ebiom.2021.103346)
Supplement: Supplementary file 1 [file mmc1.docx]

**Supplementary Table 1: Frequencies of HLA class I and II alleles in enrolled children**

| **Locus** | **Allele** | **Frequency** |
| --- | --- | --- |
| A | 1:01 | 0·12 |
| A | 1:03 | 0·00089 |
| A | 2:01 | 0·031 |
| A | 2:03 | 0·034 |
| A | 2:05 | 0·0017 |
| A | 2:06 | 0·015 |
| A | 2:11 | 0·034 |
| A | 2:16 | 0·0016 |
| A | 2:20 | 0·0017 |
| A | 3:01 | 0·047 |
| A | 3:02 | 0·0032 |
| A | 11:01 | 0’20 |
| A | 11:03 | 0·0048 |
| A | 11:12 | 0·00089 |
| A | 23:01 | 0·00079 |
| A | 24:02 | 0·12 |
| A | 24:03 | 0·0034 |
| A | 24:07 | 0·030 |
| A | 24:17 | 0·015 |
| A | 24:30 | 0·00089 |
| A | 26:01 | 0·023 |
| A | 29:01 | 0·011 |
| A | 30:01 | 0·0077 |
| A | 30:02 | 0·00079 |
| A | 31:01 | 0·025 |
| A | 31:12 | 0·00079 |
| A | 31:16 | 0·0032 |
| A | 32:01 | 0·017 |
| A | 33:00 | 0·00089 |
| A | 33:03 | 0·18 |
| A | 68:01 | 0·054 |
| A | 68:09 | 0·00079 |
| A | 74:01 | 0·0058 |
| B | 7:02 | 0·030 |
| B | 7:05 | 0·017 |
| B | 8:01 | 0·0074 |
| B | 13:01 | 0·024 |
| B | 13:02 | 0·0093 |
| B | 14:02 | 0·00089 |
| B | 15:00 | 0·0018 |
| B | 15:01 | 0·013 |
| B | 15:02 | 0·11 |
| B | 15:05 | 0·0042 |
| B | 15:07 | 0·00079 |
| B | 15:08 | 0·022 |
| B | 15:10 | 0·00079 |
| B | 15:12 | 0·0027 |
| B | 15:17 | 0·011 |
| B | 15:18 | 0·0057 |
| B | 15:25 | 0·016 |
| B | 15:32 | 0·017 |
| B | 18:01 | 0·011 |
| B | 27:04 | 0·00089 |
| B | 27:05 | 0·032 |
| B | 27:07 | 0·0049 |
| B | 27:61 | 0·0018 |
| B | 35:00 | 0·00079 |
| B | 35:01 | 0·050 |
| B | 35:03 | 0·064 |
| B | 35:05 | 0·0090 |
| B | 35:08 | 0·0059 |
| B | 35:13 | 0·0017 |
| B | 37:01 | 0·026 |
| B | 38:01 | 0·00079 |
| B | 38:02 | 0·036 |
| B | 39:01 | 0·0059 |
| B | 39:06 | 0·0017 |
| B | 40:00 | 0·00089 |
| B | 40:01 | 0·011 |
| B | 40:02 | 0·00089 |
| B | 40:06 | 0·048 |
| B | 40:23 | 0·00089 |
| B | 40:26 | 0·00089 |
| B | 41:02 | 0·00079 |
| B | 44:02 | 0·0027 |
| B | 44:03 | 0·13 |
| B | 44:06 | 0·00089 |
| B | 45:01 | 0·00089 |
| B | 48:01 | 0·0026 |
| B | 49:01 | 0·0025 |
| B | 50:01 | 0·0017 |
| B | 51:01 | 0·034 |
| B | 51:02 | 0·0025 |
| B | 51:06 | 0·0058 |
| B | 52:01 | 0·095 |
| B | 52:04 | 0·00089 |
| B | 55:01 | 0·012 |
| B | 56:01 | 0·0060 |
| B | 57:00 | 0·00079 |
| B | 57:01 | 0·066 |
| B | 58:01 | 0·037 |
| DRB1 | 1:01 | 0·026 |
| DRB1 | 3:01 | 0·031 |
| DRB1 | 4:01 | 0·012 |
| DRB1 | 4:02 | 0·0033 |
| DRB1 | 4:03 | 0·053 |
| DRB1 | 4:04 | 0·0083 |
| DRB1 | 4:05 | 0·016 |
| DRB1 | 4:06 | 0·0050 |
| DRB1 | 4:08 | 0·0017 |
| DRB1 | 7:01 | 0·25 |
| DRB1 | 7:03 | 0·0025 |
| DRB1 | 8:01 | 0·0018 |
| DRB1 | 8:02 | 0·00079 |
| DRB1 | 8:03 | 0·0034 |
| DRB1 | 9:01 | 0·0093 |
| DRB1 | 10:01 | 0·063 |
| DRB1 | 11:01 | 0·024 |
| DRB1 | 11:04 | 0·00089 |
| DRB1 | 11:06 | 0·00089 |
| DRB1 | 11:08 | 0·0017 |
| DRB1 | 11:11 | 0·00079 |
| DRB1 | 12:01 | 0·0051 |
| DRB1 | 12:02 | 0·091 |
| DRB1 | 13:01 | 0·022 |
| DRB1 | 13:02 | 0·012 |
| DRB1 | 14:01 | 0·0069 |
| DRB1 | 14:04 | 0·041 |
| DRB1 | 14:05 | 0·00089 |
| DRB1 | 14:07 | 0·00079 |
| DRB1 | 14:10 | 0·00079 |
| DRB1 | 14:19 | 0·00079 |
| DRB1 | 14:28 | 0·00089 |
| DRB1 | 15:01 | 0·090 |
| DRB1 | 15:02 | 0·17 |
| DRB1 | 15:04 | 0·013 |
| DRB1 | 15:06 | 0·012 |
| DRB1 | 15:20 | 0·00089 |
| DRB1 | 16:01 | 0·00079 |
| DRB1 | 16:02 | 0·010 |
| DQA1 | 1:01 | 0·19 |
| DQA1 | 1:02 | 0·12 |
| DQA1 | 1:03 | 0·17 |
| DQA1 | 2:01 | 0·26 |
| DQA1 | 3:01 | 0·11 |
| DQA1 | 4:01 | 0·0026 |
| DQA1 | 5:01 | 0·066 |
| DQA1 | 6:01 | 0·088 |
| DQB1 | 2:01 | 0·20 |
| DQB1 | 3:00 | 0·00089 |
| DQB1 | 3:01 | 0·13 |
| DQB1 | 3:02 | 0·080 |
| DQB1 | 3:03 | 0·096 |
| DQB1 | 3:05 | 0·00089 |
| DQB1 | 4:01 | 0·0067 |
| DQB1 | 4:02 | 0·011 |
| DQB1 | 5:00 | 0·00079 |
| DQB1 | 5:01 | 0·13 |
| DQB1 | 5:02 | 0·060 |
| DQB1 | 5:03 | 0·067 |
| DQB1 | 6:00 | 0·00079 |
| DQB1 | 6:01 | 0·17 |
| DQB1 | 6:02 | 0·014 |
| DQB1 | 6:03 | 0·023 |
| DQB1 | 6:04 | 0·0092 |
| DQB1 | 6:09 | 0·0033 |

**Supplementary Table 2: Novel HLA alleles**

For analysis HLA allele names must conform to the colon-delimited allele name nomenclature as defined by the WHO Nomenclature Committee for Factors of the HLA System in April 2010. Of 3005 alleles genotyped (601 subjects genotyped for 5 loci), nine novel alleles were discovered in the dataset. The word “new” was replaced with “00” for purposes of analysis; however, those alleles with the designation “00” in the second field did not include all known alleles of the serotype but rather represented an allele with a unique coding sequence not previously described. The two alleles designated B*15:00 do not represent the same sequence.

| **Original format** | **BIGDAWG format** | **SIDs changed** |
| --- | --- | --- |
| A*33:new | 33:00 | 1691 |
| B*15:new | 15:00 | 1111 |
| B*15:new | 15:00 | 1499 |
| B*35:new | 35:00 | 1317 |
| B*40:new | 40:00 | 1359 |
| B*57:new | 57:00 | 1575 |
| DQB1*03:new | 03:00 | 1431 |
| DQB1*05:new | 05:00 | 1557 |
| DQB1*06:new | 06:00 | 1207 |

**Supplementary Table 3: HLA class I and II allele-pathogen associations**

| **Pathogen** | **Allele** | **Controls** | **Cases** | **Frequency (Controls)** | **Frequency (Cases)** | **P-value_Chi-square value** | **Chi-square value** | **Degrees of freedom** | **Odds-Ratio** | **Confidence Interval Lower 95%** | **Confidence Interval Upper 95%** | **P-value** |
| --- | --- | --- | --- | --- | --- | --- | --- | --- | --- | --- | --- | --- |
| EAEC | *A*24:02* | 50 | 94 | 0·17 | 0·10 | 0·11 | 18 | 12 | 0·55 | 0·38 | 0·82 | 0·0015 |
|  | *B*57:01* | 12 | 68 | 0·041 | 0·075 | 0·36 | 17 | 16 | 1·9 | 0·98 | 3·8 | 0·048 |
|  | *DQA1*01:03* | 38 | 166 | 0·13 | 0·18 | 0·51 | 6·2 | 7·0 | 1·5 | 1·0 | 2·2 | 0·044 |
|  | *DRB1*14:04* | 19 | 30 | 0·066 | 0·033 | 0·76 | 5·8 | 9·0 | 0·49 | 0·26 | 0·93 | 0·015 |
| Adenovirus | *A*24:02* | 74 | 70 | 0·15 | 0·10 | 0·10 | 24 | 16 | 0·64 | 0·45 | 0·93 | 0·013 |
|  | *B*27:05* | 22 | 16 | 0·044 | 0·023 | 0·33 | 25 | 23 | 0·51 | 0·25 | 1·0 | 0·040 |
| LT-ETEC | *A*02:11* | 24 | 16 | 0·046 | 0·024 | 0·057 | 26 | 16 | 0·5 | 0·25 | 0·99 | 0·032 |
| ST-ETEC | *A*26:01* | 8 | 20 | 0·014 | 0·031 | 0·057 | 26 | 16 | 2·3 | 0·95 | 6·0 | 0·047 |
|  | *B*57:01* | 29 | 51 | 0·051 | 0·080 | 0·40 | 24 | 23 | 1·6 | 0·99 | 2·7 | 0·044 |
| EPEC | *A*02:03* | 26 | 14 | 0·045 | 0·022 | 0·19 | 21 | 16 | 0·49 | 0·23 | 0·98 | 0·029 |
|  | *A*24:17* | 3 | 15 | 0·0052 | 0·024 | 0·19 | 21 | 16 | 4·7 | 1·3 | 26 | 0·0072 |
|  | *B*35:05* | 1 | 10 | 0·0017 | 0·016 | 0·25 | 29 | 25 | 9·4 | 1·3 | 408 | 0·0093 |
|  | *DQB1*04:02* | 10 | 3 | 0·017 | 0·0048 | 0·57 | 10 | 12 | 0·27 | 0·050 | 1·1 | 0·036 |
|  | *DRB1*15:01* | 42 | 67 | 0·073 | 0·11 | 0·61 | 16 | 18 | 1·5 | 1·0 | 2·4 | 0·035 |
| Rotavirus | *B*37:01* | 9 | 2 | 0·030 | 0·0067 | 0·18 | 21 | 16 | 0·22 | 0·020 | 1·1 | 0·035 |
| *C· jejuni/coli* | *A*29:01* | 11 | 2 | 0·016 | 0·0038 | 0·46 | 16 | 16 | 0·23 | 0·02 | 1·0 | 0·035 |
|  | *B*35:01* | 26 | 34 | 0·039 | 0·064 | 0·61 | 21 | 23 | 1·7 | 0·97 | 3·0 | 0·047 |
|  | *B*38:02* | 16 | 27 | 0·024 | 0·051 | 0·61 | 21 | 23 | 2·2 | 1·1 | 4·4 | 0·013 |
| Sapovirus | *A*01:01* | 71 | 73 | 0·10 | 0·14 | 0·053 | 26 | 16 | 1·4 | 1·0 | 2·1 | 0·040 |
|  | *A*02:11* | 30 | 10 | 0·044 | 0·019 | 0·053 | 26 | 16 | 0·44 | 0·19 | 0·93 | 0·021 |
|  | *A*32:01* | 16 | 4 | 0·023 | 0·0078 | 0·053 | 26 | 16 | 0·33 | 0·080 | 1·0 | 0·038 |
| Astrovirus | *A*24:02* | 105 | 39 | 0·13 | 0·094 | 0·41 | 16 | 15 | 0·68 | 0·45 | 1·0 | 0·048 |
|  | *B*15:01* | 5 | 10 | 0·0064 | 0·024 | 0·49 | 18 | 19 | 3·9 | 1·2 | 15 | 0·0082 |
|  | *B*38:02* | 20 | 23 | 0·025 | 0·056 | 0·49 | 18 | 19 | 2·3 | 1·2 | 4·4 | 0·0074 |
| *Cryptosporidium* | *B*38:02* | 29 | 14 | 0·029 | 0·069 | 0·069 | 21 | 13 | 2·5 | 1·2 | 4·9 | 0·0056 |
|  | *DQA1*01:01* | 178 | 51 | 0·18 | 0·25 | 0·23 | 9·3 | 7·0 | 1·5 | 1·1 | 2·2 | 0·018 |
|  | *DQB1*05:01* | 124 | 36 | 0·12 | 0·18 | 0·53 | 7·0 | 8·0 | 1·5 | 0·98 | 2·3 | 0·045 |

**Supplementary Table 4: Five-locus haplotype frequencies observed in the data set**

| A~B~DRB1~DQA1~DQB1 | Frequency |
| --- | --- |
| 01:01~07:02~04:03~03:01~03:02 | 0·0025 |
| 01:01~07:02~07:01~02:01~02:01 | 0·00088 |
| 01:01~07:02~15:01~01:02~06:02 | 0·0016 |
| 01:01~07:05~15:01~01:03~06:01 | 0·00079 |
| 01:01~13:01~14:04~01:01~05:03 | 0·0017 |
| 01:01~13:01~15:01~01:02~06:01 | 0·0016 |
| 01:01~14:02~12:02~06:01~06:09 | 0·00088 |
| 01:01~15:02~04:06~03:01~03:02 | 0·00079 |
| 01:01~15:02~15:04~01:03~05:02 | 0·00079 |
| 01:01~15:02~15:06~01:02~05:02 | 0·00088 |
| 01:01~15:08~07:01~02:01~03:03 | 0·0016 |
| 01:01~15:17~01:01~01:01~05:01 | 0·00079 |
| 01:01~15:17~03:01~05:01~02:01 | 0·00079 |
| 01:01~15:17~07:01~02:01~02:01 | 0·00079 |
| 01:01~15:17~12:02~01:02~05:02 | 0·00079 |
| 01:01~15:17~13:02~01:02~06:00 | 0·00079 |
| 01:01~15:17~13:02~01:02~06:03 | 0·00079 |
| 01:01~15:17~13:02~01:02~06:04 | 0·0017 |
| 01:01~15:25~08:03~06:01~03:01 | 0·00088 |
| 01:01~15:32~12:02~01:02~03:02 | 0·00088 |
| 01:01~27:05~01:01~01:01~05:01 | 0·0017 |
| 01:01~27:05~04:04~03:01~03:02 | 0·0025 |
| 01:01~27:05~13:01~01:03~06:03 | 0·00079 |
| 01:01~27:05~14:04~01:01~05:03 | 0·0017 |
| 01:01~35:01~07:01~02:01~02:01 | 0·00088 |
| 01:01~35:01~10:01~01:01~05:01 | 0·00088 |
| 01:01~35:03~04:03~03:01~03:02 | 0·00079 |
| 01:01~35:03~07:01~02:01~03:03 | 0·00088 |
| 01:01~35:03~13:01~01:03~06:03 | 0·0018 |
| 01:01~35:03~14:01~01:01~05:03 | 0·00088 |
| 01:01~35:03~15:01~01:02~06:01 | 0·00079 |
| 01:01~35:03~15:02~01:03~06:01 | 0·0016 |
| 01:01~37:01~01:01~01:01~05:01 | 0·00079 |
| 01:01~37:01~10:01~01:01~05:01 | 0·012 |
| 01:01~38:02~15:01~01:02~06:01 | 0·0026 |
| 01:01~44:03~07:01~02:01~02:01 | 0·0068 |
| 01:01~44:03~12:02~06:01~03:01 | 0·00079 |
| 01:01~51:01~04:03~03:01~03:02 | 0·00079 |
| 01:01~51:01~07:01~02:01~02:01 | 0·0026 |
| 01:01~51:01~07:01~02:01~03:03 | 0·0017 |
| 01:01~52:01~04:03~03:01~03:02 | 0·00088 |
| 01:01~52:01~07:01~02:01~02:01 | 0·00079 |
| 01:01~52:01~13:02~01:02~03:01 | 0·00088 |
| 01:01~52:01~15:01~01:03~06:01 | 0·00079 |
| 01:01~52:01~15:02~01:03~06:01 | 0·0017 |
| 01:01~55:01~08:03~06:01~03:01 | 0·00079 |
| 01:01~56:01~15:02~01:03~06:01 | 0·00088 |
| 01:01~57:01~04:01~03:01~03:02 | 0·00088 |
| 01:01~57:01~07:01~02:01~02:01 | 0·0050 |
| 01:01~57:01~07:01~02:01~03:03 | 0·031 |
| 01:01~57:01~09:01~03:01~03:03 | 0·00088 |
| 01:01~57:01~12:01~06:01~03:01 | 0·00079 |
| 01:01~57:01~12:02~06:01~03:01 | 0·0017 |
| 01:01~57:01~15:01~01:01~05:01 | 0·00079 |
| 01:01~57:01~15:02~01:03~06:01 | 0·0024 |
| 01:01~57:01~16:02~01:02~05:02 | 0·00088 |
| 01:01~58:01~03:01~05:01~02:01 | 0·0025 |
| 01:01~58:01~15:01~01:02~06:02 | 0·00079 |
| 01:03~35:01~14:04~01:01~05:03 | 0·00088 |
| 02:01~07:02~15:01~01:02~06:01 | 0·00088 |
| 02:01~07:02~15:01~01:02~06:02 | 0·0026 |
| 02:01~07:02~15:02~01:01~05:01 | 0·00088 |
| 02:01~13:01~08:01~04:01~04:02 | 0·00088 |
| 02:01~13:02~07:01~02:01~02:01 | 0·00079 |
| 02:01~13:02~10:01~01:01~05:01 | 0·00088 |
| 02:01~15:01~14:04~01:01~06:01 | 0·00079 |
| 02:01~15:02~12:02~06:01~03:01 | 0·0016 |
| 02:01~15:08~04:03~03:01~03:02 | 0·00088 |
| 02:01~15:17~15:02~01:03~06:01 | 0·00088 |
| 02:01~15:18~07:01~02:01~03:03 | 0·00088 |
| 02:01~18:01~04:05~03:01~04:02 | 0·00079 |
| 02:01~18:01~07:01~02:01~03:03 | 0·00088 |
| 02:01~27:05~04:05~05:01~04:01 | 0·00088 |
| 02:01~27:05~14:01~01:01~06:01 | 0·00088 |
| 02:01~27:05~15:01~01:02~06:02 | 0·00079 |
| 02:01~35:01~11:01~05:01~03:01 | 0·00079 |
| 02:01~35:01~15:02~01:01~05:03 | 0·00079 |
| 02:01~35:03~07:01~02:01~02:01 | 0·00088 |
| 02:01~35:03~13:02~01:02~06:04 | 0·00079 |
| 02:01~35:03~15:01~01:02~06:01 | 0·00079 |
| 02:01~38:02~07:01~01:01~05:02 | 0·00088 |
| 02:01~40:01~15:01~01:01~05:01 | 0·00079 |
| 02:01~40:01~15:01~01:02~06:02 | 0·00079 |
| 02:01~40:06~04:01~03:01~03:02 | 0·00088 |
| 02:01~40:06~15:20~01:01~05:03 | 0·00088 |
| 02:01~40:23~09:01~03:01~03:02 | 0·00088 |
| 02:01~44:03~01:01~01:01~05:01 | 0·00079 |
| 02:01~44:03~07:01~02:01~02:01 | 0·00079 |
| 02:01~44:03~15:01~01:03~05:02 | 0·00088 |
| 02:01~51:01~04:04~03:01~03:02 | 0·0018 |
| 02:01~55:01~07:01~02:01~02:01 | 0·00079 |
| 02:01~58:01~13:02~01:02~06:09 | 0·00088 |
| 02:03~07:02~07:01~02:01~02:01 | 0·00079 |
| 02:03~13:01~15:02~01:01~05:01 | 0·00079 |
| 02:03~13:01~16:02~01:02~05:02 | 0·0018 |
| 02:03~15:02~07:01~02:01~02:01 | 0·00079 |
| 02:03~15:02~12:02~06:01~03:01 | 0·00079 |
| 02:03~15:02~15:02~01:01~05:01 | 0·011 |
| 02:03~15:25~04:05~03:01~04:01 | 0·00079 |
| 02:03~15:25~12:02~06:01~03:01 | 0·00079 |
| 02:03~15:25~15:01~01:02~05:02 | 0·00079 |
| 02:03~27:05~01:01~01:01~03:02 | 0·00088 |
| 02:03~35:01~04:08~03:01~03:01 | 0·00088 |
| 02:03~38:02~15:01~01:02~06:01 | 0·00088 |
| 02:03~38:02~15:02~01:01~05:01 | 0·0059 |
| 02:03~38:02~15:02~01:01~05:02 | 0·00088 |
| 02:03~39:01~04:05~03:01~04:01 | 0·0016 |
| 02:03~40:01~15:02~01:01~05:01 | 0·00088 |
| 02:03~44:02~12:02~01:02~06:01 | 0·00088 |
| 02:03~44:03~15:02~01:03~06:01 | 0·00088 |
| 02:03~51:01~15:06~01:02~05:02 | 0·00088 |
| 02:03~57:01~07:01~02:01~03:03 | 0·00088 |
| 02:05~40:06~15:01~01:03~06:04 | 0·00088 |
| 02:05~50:01~07:01~02:01~02:01 | 0·00079 |
| 02:06~07:02~10:01~01:01~05:01 | 0·00079 |
| 02:06~15:01~04:06~03:01~03:02 | 0·00079 |
| 02:06~15:01~15:02~01:03~06:01 | 0·00079 |
| 02:06~15:02~15:01~01:02~06:01 | 0·0025 |
| 02:06~15:25~15:02~01:01~05:01 | 0·00088 |
| 02:06~27:05~15:02~01:01~05:02 | 0·00079 |
| 02:06~35:01~15:06~01:02~05:02 | 0·00079 |
| 02:06~35:03~12:01~05:01~03:01 | 0·0018 |
| 02:06~35:03~13:01~01:03~06:03 | 0·00079 |
| 02:06~37:01~14:04~02:01~03:03 | 0·00079 |
| 02:06~40:06~15:01~01:03~06:01 | 0·00088 |
| 02:06~44:03~15:01~01:02~06:01 | 0·00079 |
| 02:06~52:01~07:01~02:01~03:03 | 0·00088 |
| 02:06~52:01~12:02~06:01~03:01 | 0·00079 |
| 02:06~55:01~10:01~01:01~05:01 | 0·00088 |
| 02:11~07:02~15:02~01:03~06:01 | 0·0026 |
| 02:11~13:01~12:02~06:01~03:01 | 0·00088 |
| 02:11~13:01~15:01~01:02~06:02 | 0·00079 |
| 02:11~15:01~13:01~01:03~06:03 | 0·00088 |
| 02:11~15:05~10:01~01:01~05:01 | 0·00088 |
| 02:11~15:05~13:01~01:03~06:03 | 0·00088 |
| 02:11~15:05~14:04~01:01~05:03 | 0·00079 |
| 02:11~15:07~12:02~06:01~03:01 | 0·00079 |
| 02:11~15:08~04:03~03:01~03:02 | 0·00079 |
| 02:11~15:12~10:01~01:01~05:01 | 0·00088 |
| 02:11~15:17~13:02~01:02~06:04 | 0·00088 |
| 02:11~18:01~04:01~03:01~03:02 | 0·00079 |
| 02:11~27:05~15:02~01:03~06:01 | 0·00079 |
| 02:11~35:03~10:01~01:01~05:01 | 0·0018 |
| 02:11~35:03~15:02~01:03~05:02 | 0·00088 |
| 02:11~35:03~16:02~01:02~05:02 | 0·00079 |
| 02:11~40:01~09:01~03:01~03:03 | 0·00088 |
| 02:11~40:06~04:01~03:01~03:02 | 0·00088 |
| 02:11~40:06~07:01~01:02~05:02 | 0·00079 |
| 02:11~40:06~07:01~02:01~02:01 | 0·00079 |
| 02:11~40:06~14:04~01:01~05:03 | 0·00088 |
| 02:11~40:06~15:01~01:02~06:01 | 0·00088 |
| 02:11~40:06~15:01~01:03~06:01 | 0·0016 |
| 02:11~44:03~07:01~02:01~02:01 | 0·0042 |
| 02:11~49:01~13:02~01:02~06:04 | 0·00088 |
| 02:11~50:01~04:03~03:01~03:02 | 0·00088 |
| 02:11~52:01~04:06~03:01~03:02 | 0·00079 |
| 02:11~52:01~14:28~01:03~05:03 | 0·00088 |
| 02:11~52:01~15:02~01:03~06:01 | 0·00088 |
| 02:11~56:01~04:05~03:01~04:01 | 0·00088 |
| 02:11~57:01~07:01~02:01~03:03 | 0·00079 |
| 02:11~58:01~07:01~02:01~03:03 | 0·00088 |
| 02:16~07:05~15:02~01:03~06:01 | 0·00079 |
| 02:16~51:01~04:01~03:01~03:02 | 0·00079 |
| 02:20~40:02~15:02~01:03~05:03 | 0·00088 |
| 02:20~57:01~07:01~02:01~03:03 | 0·00079 |
| 03:01~07:05~15:02~01:01~05:02 | 0·00088 |
| 03:01~08:01~03:01~05:01~02:01 | 0·00079 |
| 03:01~15:01~14:04~01:01~05:03 | 0·00079 |
| 03:01~15:02~15:06~01:02~05:02 | 0·00088 |
| 03:01~15:25~15:01~01:02~05:02 | 0·00088 |
| 03:01~18:01~15:02~01:03~05:01 | 0·00079 |
| 03:01~27:05~04:04~03:01~03:02 | 0·00088 |
| 03:01~27:05~09:01~03:01~03:03 | 0·0041 |
| 03:01~35:01~01:01~01:01~05:01 | 0·0025 |
| 03:01~35:01~04:03~03:01~03:02 | 0·0016 |
| 03:01~35:01~07:01~02:01~02:01 | 0·0018 |
| 03:01~35:01~07:01~02:01~03:03 | 0·00088 |
| 03:01~35:01~13:01~01:03~06:03 | 0·00088 |
| 03:01~35:01~14:07~01:01~05:03 | 0·00079 |
| 03:01~35:01~15:02~01:03~06:01 | 0·0025 |
| 03:01~35:03~07:01~02:01~02:01 | 0·00079 |
| 03:01~35:03~11:08~05:01~03:01 | 0·00088 |
| 03:01~35:03~14:01~01:01~05:03 | 0·00079 |
| 03:01~35:03~15:02~01:03~06:01 | 0·0017 |
| 03:01~37:01~10:01~01:01~05:01 | 0·00079 |
| 03:01~40:01~04:03~03:01~03:02 | 0·0017 |
| 03:01~40:01~15:02~01:01~05:01 | 0·00088 |
| 03:01~40:06~04:03~03:01~03:02 | 0·00079 |
| 03:01~40:06~07:01~02:01~03:03 | 0·00088 |
| 03:01~40:06~15:01~01:03~06:01 | 0·0026 |
| 03:01~44:03~11:01~05:01~03:01 | 0·00088 |
| 03:01~44:03~15:01~01:02~06:01 | 0·00088 |
| 03:01~51:01~04:03~03:01~03:02 | 0·0016 |
| 03:01~51:01~10:01~01:01~05:01 | 0·00079 |
| 03:01~51:01~13:01~01:03~06:03 | 0·00079 |
| 03:01~52:01~04:03~03:01~03:02 | 0·0017 |
| 03:01~52:01~07:01~01:01~05:03 | 0·00079 |
| 03:01~52:01~07:01~02:01~02:01 | 0·00088 |
| 03:01~52:01~12:02~06:01~03:01 | 0·00088 |
| 03:01~52:01~15:01~01:02~06:01 | 0·00088 |
| 03:01~52:01~15:02~01:03~06:01 | 0·00079 |
| 03:01~56:01~15:02~01:03~06:01 | 0·00088 |
| 03:01~57:01~07:01~02:01~03:03 | 0·0032 |
| 03:02~15:18~15:02~01:03~06:01 | 0·00079 |
| 03:02~35:03~14:04~01:01~05:03 | 0·00088 |
| 03:02~57:01~01:01~01:01~05:01 | 0·00079 |
| 03:02~58:01~04:04~03:01~03:02 | 0·00079 |
| 11:01~07:02~04:03~03:01~03:05 | 0·00088 |
| 11:01~07:02~15:02~01:03~05:03 | 0·0018 |
| 11:01~13:01~04:05~03:01~04:01 | 0·0018 |
| 11:01~13:01~04:05~03:01~04:02 | 0·00079 |
| 11:01~13:01~15:01~01:02~06:01 | 0·0017 |
| 11:01~13:01~15:04~01:02~05:02 | 0·00088 |
| 11:01~15:00~01:01~01:01~05:01 | 0·00088 |
| 11:01~15:01~04:03~03:01~03:02 | 0·00088 |
| 11:01~15:01~12:02~06:01~03:00 | 0·00088 |
| 11:01~15:01~14:04~01:01~05:03 | 0·00088 |
| 11:01~15:01~15:06~01:02~05:02 | 0·0017 |
| 11:01~15:02~07:01~02:01~02:01 | 0·00088 |
| 11:01~15:02~12:02~06:01~03:01 | 0·032 |
| 11:01~15:02~13:01~01:03~06:03 | 0·00088 |
| 11:01~15:02~14:01~01:01~05:03 | 0·0018 |
| 11:01~15:02~15:01~01:02~06:01 | 0·0088 |
| 11:01~15:02~15:01~01:03~06:01 | 0·00088 |
| 11:01~15:02~15:02~01:02~05:02 | 0·00079 |
| 11:01~15:02~15:02~01:03~05:03 | 0·00088 |
| 11:01~15:02~15:02~01:03~06:01 | 0·0017 |
| 11:01~15:05~14:04~01:01~05:03 | 0·00088 |
| 11:01~15:18~13:02~02:01~02:01 | 0·00088 |
| 11:01~15:25~15:01~01:03~06:01 | 0·00079 |
| 11:01~15:25~15:04~01:02~05:02 | 0·00088 |
| 11:01~15:32~07:01~02:01~02:01 | 0·0025 |
| 11:01~15:32~07:01~02:01~03:03 | 0·0018 |
| 11:01~15:32~12:02~06:01~03:01 | 0·00079 |
| 11:01~15:32~13:01~01:03~06:03 | 0·00088 |
| 11:01~15:32~15:04~01:02~05:02 | 0·0018 |
| 11:01~18:01~13:01~01:03~06:03 | 0·00079 |
| 11:01~27:05~12:01~05:01~03:01 | 0·00088 |
| 11:01~35:00~01:01~01:01~05:01 | 0·00079 |
| 11:01~35:01~01:01~01:01~05:01 | 0·0068 |
| 11:01~35:01~03:01~05:01~02:01 | 0·00079 |
| 11:01~35:01~04:03~03:01~03:02 | 0·00088 |
| 11:01~35:01~07:01~02:01~03:03 | 0·00079 |
| 11:01~35:01~10:01~01:01~05:01 | 0·0039 |
| 11:01~35:01~11:01~05:01~03:01 | 0·0032 |
| 11:01~35:01~14:04~01:01~05:03 | 0·00079 |
| 11:01~35:01~15:02~01:01~05:01 | 0·0017 |
| 11:01~35:01~15:02~01:03~06:01 | 0·00088 |
| 11:01~35:03~03:01~05:01~02:01 | 0·00088 |
| 11:01~35:03~07:01~02:01~02:01 | 0·0024 |
| 11:01~35:03~07:01~02:01~03:03 | 0·0025 |
| 11:01~35:03~11:01~05:01~03:01 | 0·00088 |
| 11:01~35:03~12:02~06:01~03:01 | 0·0018 |
| 11:01~35:03~14:01~01:01~05:03 | 0·00088 |
| 11:01~35:03~14:10~04:01~04:02 | 0·00079 |
| 11:01~35:03~15:02~01:01~05:02 | 0·00079 |
| 11:01~35:03~15:02~01:03~06:01 | 0·00088 |
| 11:01~37:01~04:03~03:01~03:02 | 0·0017 |
| 11:01~37:01~10:01~01:01~05:01 | 0·00079 |
| 11:01~37:01~11:01~05:01~03:01 | 0·00079 |
| 11:01~37:01~12:02~06:01~03:01 | 0·00079 |
| 11:01~38:02~01:01~01:01~05:01 | 0·00079 |
| 11:01~38:02~11:01~05:01~03:01 | 0·00088 |
| 11:01~38:02~12:02~06:01~03:01 | 0·0033 |
| 11:01~38:02~15:02~01:01~05:01 | 0·0025 |
| 11:01~39:06~16:01~01:02~05:02 | 0·00079 |
| 11:01~40:00~07:01~01:02~06:04 | 0·00088 |
| 11:01~40:01~07:01~02:01~02:01 | 0·00079 |
| 11:01~40:01~11:01~05:01~03:01 | 0·00079 |
| 11:01~40:01~15:01~01:02~06:01 | 0·0016 |
| 11:01~40:06~04:02~03:01~03:02 | 0·00079 |
| 11:01~40:06~04:05~01:02~05:02 | 0·00088 |
| 11:01~40:06~07:01~02:01~03:03 | 0·00088 |
| 11:01~40:06~14:04~01:01~05:03 | 0·0033 |
| 11:01~40:06~15:02~01:03~06:01 | 0·0016 |
| 11:01~40:06~16:02~01:02~05:02 | 0·0025 |
| 11:01~44:03~04:05~03:01~04:01 | 0·00079 |
| 11:01~44:03~07:01~02:01~02:01 | 0·0075 |
| 11:01~44:03~07:01~02:01~03:03 | 0·0025 |
| 11:01~48:01~12:01~05:01~03:01 | 0·00088 |
| 11:01~48:01~16:02~01:02~05:03 | 0·00079 |
| 11:01~51:01~04:02~03:01~03:02 | 0·00079 |
| 11:01~51:01~07:01~02:01~02:01 | 0·0017 |
| 11:01~51:01~15:01~01:02~06:01 | 0·00088 |
| 11:01~51:01~15:02~01:03~06:01 | 0·0026 |
| 11:01~51:06~14:04~01:01~05:03 | 0·00079 |
| 11:01~52:01~04:01~03:01~03:01 | 0·0016 |
| 11:01~52:01~04:03~03:01~03:02 | 0·0024 |
| 11:01~52:01~11:01~05:01~03:01 | 0·0026 |
| 11:01~52:01~12:02~01:02~05:02 | 0·0017 |
| 11:01~52:01~14:04~01:01~05:03 | 0·0025 |
| 11:01~52:01~15:02~01:03~06:01 | 0·021 |
| 11:01~52:04~14:04~01:01~05:03 | 0·00088 |
| 11:01~55:01~04:03~03:01~03:02 | 0·00079 |
| 11:01~55:01~13:01~01:03~06:03 | 0·00088 |
| 11:01~55:01~15:02~01:03~06:01 | 0·0017 |
| 11:01~56:01~04:03~03:01~03:02 | 0·00079 |
| 11:01~56:01~11:01~03:01~03:01 | 0·00088 |
| 11:01~56:01~15:02~01:03~06:01 | 0·00079 |
| 11:01~57:01~07:01~02:01~03:03 | 0·0058 |
| 11:01~57:01~15:02~01:03~05:01 | 0·00079 |
| 11:01~57:01~15:02~01:03~06:01 | 0·0016 |
| 11:01~58:01~03:01~05:01~02:01 | 0·0050 |
| 11:01~58:01~04:03~03:01~03:02 | 0·00079 |
| 11:01~58:01~15:02~01:03~06:01 | 0·00079 |
| 11:03~15:02~12:02~06:01~03:01 | 0·00079 |
| 11:03~35:01~15:04~01:02~05:02 | 0·00079 |
| 11:03~38:02~14:04~01:01~05:03 | 0·00079 |
| 11:03~40:26~16:02~01:02~05:02 | 0·00088 |
| 11:03~58:01~04:01~03:01~03:01 | 0·0016 |
| 11:12~40:06~15:02~01:01~05:03 | 0·00088 |
| 23:01~58:01~13:02~01:02~06:09 | 0·00079 |
| 24:02~07:02~07:01~02:01~02:01 | 0·0059 |
| 24:02~07:02~10:01~01:01~05:01 | 0·00079 |
| 24:02~07:02~15:01~01:02~06:02 | 0·00088 |
| 24:02~07:02~15:02~01:03~06:01 | 0·00088 |
| 24:02~07:05~10:01~01:01~05:01 | 0·0016 |
| 24:02~07:05~15:01~01:03~06:01 | 0·0026 |
| 24:02~08:01~14:04~01:01~05:03 | 0·0018 |
| 24:02~13:01~07:01~02:01~03:03 | 0·00079 |
| 24:02~13:01~15:01~01:02~06:01 | 0·0017 |
| 24:02~13:01~15:02~01:03~06:01 | 0·0026 |
| 24:02~15:01~07:01~02:01~02:01 | 0·00088 |
| 24:02~15:02~04:01~03:01~03:02 | 0·00088 |
| 24:02~15:02~07:01~02:01~02:01 | 0·0031 |
| 24:02~15:02~10:01~01:03~06:01 | 0·00079 |
| 24:02~15:02~12:02~01:01~05:01 | 0·00088 |
| 24:02~15:02~12:02~05:01~03:01 | 0·00079 |
| 24:02~15:05~10:01~01:01~05:01 | 0·00079 |
| 24:02~15:08~04:03~03:01~03:02 | 0·00079 |
| 24:02~15:08~07:01~02:01~02:01 | 0·0016 |
| 24:02~15:10~04:05~03:01~05:01 | 0·00079 |
| 24:02~15:12~15:02~01:01~05:02 | 0·00088 |
| 24:02~15:25~15:01~01:02~05:02 | 0·00079 |
| 24:02~15:25~15:01~01:02~06:01 | 0·00079 |
| 24:02~15:25~15:04~01:02~05:02 | 0·00088 |
| 24:02~15:32~14:04~01:01~05:03 | 0·00088 |
| 24:02~18:01~04:06~03:01~03:02 | 0·00088 |
| 24:02~18:01~07:01~02:01~02:01 | 0·00079 |
| 24:02~18:01~15:01~01:02~06:01 | 0·00079 |
| 24:02~27:05~09:01~03:01~03:03 | 0·00088 |
| 24:02~27:07~04:06~03:01~03:02 | 0·00088 |
| 24:02~27:07~10:01~01:01~04:02 | 0·00079 |
| 24:02~27:07~15:02~02:01~02:01 | 0·00088 |
| 24:02~35:01~11:01~05:01~03:01 | 0·00088 |
| 24:02~35:01~12:02~06:01~03:01 | 0·0017 |
| 24:02~35:01~14:04~01:03~05:03 | 0·00088 |
| 24:02~35:01~15:01~01:02~06:01 | 0·0017 |
| 24:02~35:01~15:06~01:02~05:02 | 0·00088 |
| 24:02~35:03~03:01~05:01~02:01 | 0·0018 |
| 24:02~35:03~04:03~03:01~03:02 | 0·00079 |
| 24:02~35:03~07:01~02:01~02:01 | 0·00088 |
| 24:02~35:03~07:01~02:01~03:03 | 0·0051 |
| 24:02~35:03~10:01~01:01~05:01 | 0·00088 |
| 24:02~35:03~11:01~05:01~03:01 | 0·0017 |
| 24:02~35:03~13:01~01:03~06:03 | 0·0033 |
| 24:02~35:03~14:01~01:01~05:03 | 0·00079 |
| 24:02~35:03~14:04~01:01~05:03 | 0·00079 |
| 24:02~35:03~15:02~01:03~06:01 | 0·00088 |
| 24:02~35:08~07:01~02:01~02:01 | 0·00088 |
| 24:02~35:08~15:02~01:03~05:03 | 0·00079 |
| 24:02~35:13~13:01~01:03~06:03 | 0·00079 |
| 24:02~37:01~07:01~02:01~02:01 | 0·00088 |
| 24:02~38:02~15:02~01:01~05:01 | 0·0048 |
| 24:02~38:02~15:02~01:03~05:02 | 0·0017 |
| 24:02~39:06~08:01~04:01~04:02 | 0·00088 |
| 24:02~40:01~15:02~01:03~05:03 | 0·00079 |
| 24:02~40:06~07:01~02:01~02:01 | 0·00088 |
| 24:02~40:06~07:01~02:01~03:03 | 0·00088 |
| 24:02~40:06~14:04~01:01~05:03 | 0·0059 |
| 24:02~40:06~15:01~01:02~06:01 | 0·0016 |
| 24:02~40:06~15:01~01:03~06:01 | 0·0017 |
| 24:02~40:06~15:02~01:03~06:01 | 0·0032 |
| 24:02~44:03~07:01~02:01~02:01 | 0·0018 |
| 24:02~51:01~04:01~03:01~03:02 | 0·00088 |
| 24:02~51:01~04:05~03:01~04:02 | 0·00088 |
| 24:02~51:01~10:01~01:01~05:01 | 0·00088 |
| 24:02~51:01~15:01~01:02~06:02 | 0·00079 |
| 24:02~51:01~15:01~01:03~05:01 | 0·00088 |
| 24:02~51:02~07:01~02:01~03:03 | 0·00088 |
| 24:02~51:02~12:02~06:01~03:01 | 0·00079 |
| 24:02~51:06~12:02~06:01~03:01 | 0·00088 |
| 24:02~51:06~14:05~01:01~05:03 | 0·00088 |
| 24:02~51:06~15:01~01:02~05:02 | 0·00088 |
| 24:02~52:01~04:01~03:01~03:01 | 0·00079 |
| 24:02~52:01~04:01~03:01~03:02 | 0·00088 |
| 24:02~52:01~04:03~03:01~03:02 | 0·0018 |
| 24:02~52:01~07:01~02:01~02:01 | 0·0016 |
| 24:02~52:01~11:01~05:01~03:01 | 0·00079 |
| 24:02~52:01~13:01~01:03~06:03 | 0·00088 |
| 24:02~52:01~15:02~01:03~06:01 | 0·0083 |
| 24:02~52:01~15:04~01:02~05:02 | 0·00079 |
| 24:02~55:01~07:01~02:01~03:03 | 0·0027 |
| 24:02~56:01~14:04~01:01~05:03 | 0·00088 |
| 24:02~57:01~07:01~02:01~03:03 | 0·0017 |
| 24:03~39:01~15:02~01:03~05:03 | 0·0034 |
| 24:07~07:05~15:01~01:03~06:01 | 0·00079 |
| 24:07~15:02~01:01~01:01~05:01 | 0·00079 |
| 24:07~15:02~04:05~03:01~04:02 | 0·0025 |
| 24:07~15:25~04:03~03:01~03:02 | 0·00079 |
| 24:07~15:25~14:04~01:01~05:00 | 0·00079 |
| 24:07~27:05~09:01~03:01~03:03 | 0·00088 |
| 24:07~27:05~10:01~01:01~05:01 | 0·0018 |
| 24:07~35:05~12:02~06:01~03:01 | 0·0082 |
| 24:07~35:05~15:02~01:03~06:01 | 0·00088 |
| 24:07~37:01~15:01~01:03~05:01 | 0·00088 |
| 24:07~40:06~07:01~02:01~03:03 | 0·00088 |
| 24:07~44:03~07:01~02:01~02:01 | 0·00088 |
| 24:07~52:01~04:03~03:01~03:02 | 0·0048 |
| 24:07~52:01~07:01~02:01~02:01 | 0·00079 |
| 24:07~52:01~10:01~01:01~05:01 | 0·00079 |
| 24:07~52:01~14:04~01:01~05:03 | 0·00088 |
| 24:07~52:01~15:02~01:03~05:03 | 0·00079 |
| 24:07~57:01~12:02~06:01~03:01 | 0·00079 |
| 24:07~58:01~03:01~05:01~02:01 | 0·00088 |
| 24:17~13:01~15:02~01:03~06:01 | 0·00088 |
| 24:17~15:02~12:02~06:01~03:01 | 0·0024 |
| 24:17~15:02~13:01~01:03~06:03 | 0·00088 |
| 24:17~15:08~04:04~03:01~03:02 | 0·00079 |
| 24:17~15:25~11:01~05:01~03:01 | 0·00079 |
| 24:17~15:32~15:01~01:02~06:01 | 0·00088 |
| 24:17~18:01~12:01~05:01~03:01 | 0·00079 |
| 24:17~18:01~15:02~01:03~06:01 | 0·00079 |
| 24:17~27:05~04:06~01:02~06:01 | 0·00088 |
| 24:17~35:01~15:04~01:02~05:02 | 0·00079 |
| 24:17~38:02~15:02~01:01~05:02 | 0·00079 |
| 24:17~39:01~13:01~01:03~06:03 | 0·00088 |
| 24:17~44:03~04:03~03:01~03:02 | 0·00088 |
| 24:17~52:01~04:03~03:01~03:02 | 0·00079 |
| 24:17~52:01~07:01~02:01~02:01 | 0·00079 |
| 24:17~57:01~15:01~01:02~06:01 | 0·00079 |
| 24:30~27:04~12:02~01:02~05:02 | 0·00088 |
| 26:01~07:02~15:01~01:03~06:01 | 0·00088 |
| 26:01~08:01~03:01~05:01~02:01 | 0·0016 |
| 26:01~08:01~10:01~01:01~05:01 | 0·0024 |
| 26:01~15:01~15:01~01:02~06:02 | 0·00079 |
| 26:01~15:32~12:02~06:01~03:01 | 0·00079 |
| 26:01~27:05~04:04~03:01~03:02 | 0·00079 |
| 26:01~27:05~10:01~01:01~05:01 | 0·00079 |
| 26:01~27:05~15:02~01:01~05:03 | 0·00088 |
| 26:01~27:07~14:04~01:01~05:03 | 0·00079 |
| 26:01~35:03~10:01~01:01~05:01 | 0·00079 |
| 26:01~37:01~10:01~01:01~05:01 | 0·00088 |
| 26:01~37:01~15:01~01:03~06:01 | 0·00088 |
| 26:01~38:01~11:01~05:01~03:01 | 0·00079 |
| 26:01~38:02~04:03~03:01~05:01 | 0·00088 |
| 26:01~38:02~15:02~01:01~05:01 | 0·00079 |
| 26:01~40:06~15:01~01:03~06:01 | 0·00088 |
| 26:01~44:02~15:01~01:02~06:01 | 0·00088 |
| 26:01~52:01~04:03~03:01~03:02 | 0·0016 |
| 26:01~52:01~15:02~01:03~06:01 | 0·0024 |
| 26:01~52:01~15:02~02:01~02:01 | 0·00088 |
| 26:01~55:01~01:01~01:01~05:01 | 0·00079 |
| 26:01~57:01~07:01~02:01~03:03 | 0·00079 |
| 29:01~07:02~04:03~03:01~03:02 | 0·00088 |
| 29:01~07:05~10:01~01:01~05:01 | 0·0068 |
| 29:01~07:05~14:04~01:01~05:03 | 0·00088 |
| 29:01~07:05~15:02~01:03~06:01 | 0·00088 |
| 29:01~51:01~15:02~01:02~05:02 | 0·00088 |
| 29:01~52:01~15:02~01:03~06:01 | 0·00079 |
| 30:01~13:02~07:01~02:01~02:01 | 0·0059 |
| 30:01~13:02~15:01~01:02~06:01 | 0·0018 |
| 30:02~41:02~11:01~05:01~03:01 | 0·00079 |
| 31:01~15:02~12:02~06:01~03:01 | 0·00079 |
| 31:01~15:08~04:03~03:01~03:02 | 0·0066 |
| 31:01~15:08~07:01~02:01~03:03 | 0·0043 |
| 31:01~15:08~10:01~01:01~05:01 | 0·0017 |
| 31:01~15:08~11:01~05:01~03:01 | 0·00088 |
| 31:01~15:08~15:02~01:01~05:01 | 0·00079 |
| 31:01~15:08~15:02~01:03~06:01 | 0·0017 |
| 31:01~18:01~15:02~01:01~05:02 | 0·00079 |
| 31:01~40:06~15:02~01:03~06:01 | 0·00088 |
| 31:01~44:03~07:01~02:01~02:01 | 0·00088 |
| 31:01~49:01~13:02~01:02~06:04 | 0·00079 |
| 31:01~51:01~10:01~01:01~05:01 | 0·00079 |
| 31:01~52:01~15:02~01:03~05:03 | 0·00079 |
| 31:01~52:01~15:02~01:03~06:01 | 0·0026 |
| 31:01~58:01~01:01~01:01~05:01 | 0·00079 |
| 31:12~07:02~11:08~05:01~03:01 | 0·00079 |
| 31:16~35:03~03:01~05:01~02:01 | 0·0016 |
| 31:16~35:13~15:01~03:01~03:02 | 0·00088 |
| 31:16~57:01~15:01~01:03~06:01 | 0·00079 |
| 32:01~08:01~03:01~05:01~02:01 | 0·00088 |
| 32:01~15:01~01:01~01:01~05:01 | 0·00088 |
| 32:01~15:02~07:01~02:01~03:03 | 0·00088 |
| 32:01~15:02~12:02~06:01~03:01 | 0·00088 |
| 32:01~15:02~14:04~01:01~05:03 | 0·00079 |
| 32:01~18:01~01:01~01:01~03:02 | 0·00088 |
| 32:01~27:05~04:01~03:01~03:02 | 0·00088 |
| 32:01~27:05~07:01~01:03~06:01 | 0·00088 |
| 32:01~27:05~15:01~01:02~06:01 | 0·00079 |
| 32:01~35:01~04:03~03:01~05:01 | 0·00088 |
| 32:01~35:01~15:06~01:02~06:01 | 0·00088 |
| 32:01~35:01~15:06~02:01~03:02 | 0·00079 |
| 32:01~35:03~01:01~01:01~05:01 | 0·00088 |
| 32:01~35:08~15:01~01:03~06:01 | 0·00088 |
| 32:01~37:01~15:06~01:02~05:02 | 0·00079 |
| 32:01~40:06~15:01~01:03~06:01 | 0·00079 |
| 32:01~49:01~11:11~05:01~03:01 | 0·00079 |
| 32:01~51:01~04:03~03:01~03:02 | 0·00079 |
| 32:01~51:06~15:02~01:03~06:01 | 0·00079 |
| 32:01~57:01~12:02~06:01~03:03 | 0·00079 |
| 33:00~44:03~13:02~01:02~05:02 | 0·00088 |
| 33:03~07:02~11:06~05:01~03:01 | 0·00088 |
| 33:03~07:02~15:02~01:03~06:01 | 0·00088 |
| 33:03~07:05~10:01~01:01~05:01 | 0·00079 |
| 33:03~07:05~15:01~01:03~05:03 | 0·00079 |
| 33:03~13:01~03:01~05:01~02:01 | 0·00079 |
| 33:03~13:01~12:02~06:01~03:01 | 0·0017 |
| 33:03~13:01~15:06~01:02~05:02 | 0·00079 |
| 33:03~15:01~07:01~02:01~02:01 | 0·00088 |
| 33:03~15:01~15:06~01:02~05:02 | 0·00088 |
| 33:03~15:02~04:05~03:01~04:02 | 0·0018 |
| 33:03~15:02~12:02~06:01~03:01 | 0·0042 |
| 33:03~15:02~15:01~01:02~06:01 | 0·0040 |
| 33:03~15:02~15:02~01:01~05:01 | 0·0025 |
| 33:03~15:02~16:02~01:02~05:02 | 0·00079 |
| 33:03~15:17~07:01~02:01~03:03 | 0·00079 |
| 33:03~15:17~13:01~01:03~06:03 | 0·00088 |
| 33:03~15:18~03:01~05:01~02:01 | 0·00079 |
| 33:03~15:25~04:03~05:01~02:01 | 0·00079 |
| 33:03~15:25~08:03~06:01~03:01 | 0·00088 |
| 33:03~15:32~12:02~06:01~03:01 | 0·0025 |
| 33:03~18:01~11:04~05:01~03:01 | 0·00088 |
| 33:03~27:05~04:01~03:01~03:02 | 0·00079 |
| 33:03~27:05~15:04~01:02~05:02 | 0·00088 |
| 33:03~27:07~15:02~01:03~06:01 | 0·00079 |
| 33:03~35:01~03:01~03:01~03:02 | 0·00079 |
| 33:03~35:01~07:01~02:01~03:03 | 0·0017 |
| 33:03~35:01~15:02~01:03~06:01 | 0·00079 |
| 33:03~35:03~10:01~01:01~05:01 | 0·0034 |
| 33:03~35:03~13:02~01:02~06:04 | 0·00079 |
| 33:03~35:03~15:02~01:03~06:01 | 0·0016 |
| 33:03~35:03~15:04~01:02~05:02 | 0·00079 |
| 33:03~35:03~15:06~01:02~05:02 | 0·00088 |
| 33:03~35:08~03:01~05:01~02:01 | 0·00079 |
| 33:03~35:08~10:01~01:01~05:01 | 0·00088 |
| 33:03~35:08~13:01~01:03~06:03 | 0·00079 |
| 33:03~35:08~15:02~01:01~05:01 | 0·00088 |
| 33:03~37:01~10:01~01:01~05:01 | 0·00079 |
| 33:03~38:02~04:03~03:01~03:02 | 0·00088 |
| 33:03~38:02~07:01~02:01~02:01 | 0·00079 |
| 33:03~38:02~11:01~05:01~05:02 | 0·00079 |
| 33:03~38:02~15:02~01:01~05:01 | 0·0025 |
| 33:03~40:01~15:02~01:03~06:01 | 0·00079 |
| 33:03~40:06~15:01~01:02~06:04 | 0·00088 |
| 33:03~40:06~15:01~01:03~05:02 | 0·00079 |
| 33:03~40:06~15:02~01:03~06:01 | 0·00088 |
| 33:03~44:02~07:01~02:01~03:03 | 0·00088 |
| 33:03~44:03~01:01~01:01~05:01 | 0·0026 |
| 33:03~44:03~03:01~05:01~02:01 | 0·00079 |
| 33:03~44:03~04:03~03:01~03:02 | 0·00088 |
| 33:03~44:03~07:01~02:01~02:01 | 0·071 |
| 33:03~44:03~07:01~02:01~03:03 | 0·0017 |
| 33:03~44:03~07:03~02:01~02:01 | 0·0025 |
| 33:03~44:03~09:01~03:01~03:03 | 0·00079 |
| 33:03~44:03~10:01~01:01~05:01 | 0·0017 |
| 33:03~44:03~11:01~05:01~03:01 | 0·00079 |
| 33:03~44:03~13:02~01:02~06:09 | 0·00079 |
| 33:03~44:03~14:04~01:01~05:03 | 0·0017 |
| 33:03~44:03~15:01~01:02~06:02 | 0·00088 |
| 33:03~44:03~15:02~01:03~06:01 | 0·0048 |
| 33:03~48:01~03:01~05:01~02:01 | 0·00088 |
| 33:03~51:01~04:02~03:01~03:02 | 0·0018 |
| 33:03~51:01~04:03~03:01~03:02 | 0·00079 |
| 33:03~51:01~14:04~01:01~05:03 | 0·0018 |
| 33:03~51:06~15:01~01:02~05:02 | 0·0016 |
| 33:03~52:01~04:08~03:01~03:02 | 0·00079 |
| 33:03~52:01~07:01~02:01~05:02 | 0·00088 |
| 33:03~52:01~15:06~01:02~05:02 | 0·00079 |
| 33:03~52:01~16:02~01:02~05:02 | 0·0017 |
| 33:03~55:01~15:02~01:03~06:04 | 0·00079 |
| 33:03~57:00~07:01~02:01~03:03 | 0·00079 |
| 33:03~57:01~15:04~01:02~03:01 | 0·00079 |
| 33:03~58:01~03:01~05:01~02:01 | 0·0092 |
| 33:03~58:01~07:01~02:01~02:01 | 0·0041 |
| 33:03~58:01~07:01~02:01~03:03 | 0·00088 |
| 33:03~58:01~11:01~05:01~03:01 | 0·00079 |
| 33:03~58:01~13:01~01:03~06:03 | 0·00079 |
| 33:03~58:01~15:01~01:02~06:02 | 0·00079 |
| 33:03~58:01~15:02~01:03~05:03 | 0·0017 |
| 33:03~58:01~15:02~01:03~06:01 | 0·0024 |
| 68:01~07:02~15:01~01:02~06:02 | 0·00079 |
| 68:01~13:01~12:02~06:01~03:01 | 0·00088 |
| 68:01~15:00~14:04~01:01~05:03 | 0·00088 |
| 68:01~15:02~12:02~06:01~03:01 | 0·0043 |
| 68:01~15:02~15:02~01:03~06:01 | 0·00079 |
| 68:01~15:12~15:02~01:01~05:02 | 0·00088 |
| 68:01~15:17~15:02~01:03~06:01 | 0·00079 |
| 68:01~15:18~10:01~01:01~05:01 | 0·0024 |
| 68:01~15:25~08:03~06:01~03:01 | 0·00088 |
| 68:01~15:25~15:02~01:01~05:02 | 0·00079 |
| 68:01~15:25~15:04~01:02~05:02 | 0·00088 |
| 68:01~15:32~15:02~01:03~06:01 | 0·0025 |
| 68:01~15:32~15:04~01:02~05:02 | 0·00088 |
| 68:01~27:05~04:03~03:01~03:02 | 0·0027 |
| 68:01~27:05~14:19~05:01~03:01 | 0·00079 |
| 68:01~27:61~15:01~01:01~05:01 | 0·0018 |
| 68:01~35:03~07:01~02:01~02:01 | 0·00079 |
| 68:01~35:03~13:01~01:03~06:03 | 0·0017 |
| 68:01~35:03~14:01~01:01~05:03 | 0·00088 |
| 68:01~35:03~14:04~01:01~05:03 | 0·00079 |
| 68:01~35:03~15:01~01:02~06:02 | 0·00079 |
| 68:01~37:01~10:01~01:01~05:01 | 0·0025 |
| 68:01~40:01~13:01~01:03~06:03 | 0·00088 |
| 68:01~40:06~11:01~05:01~03:01 | 0·0017 |
| 68:01~40:06~15:02~01:03~05:03 | 0·00079 |
| 68:01~40:06~15:02~01:03~06:01 | 0·00079 |
| 68:01~44:03~07:01~02:01~02:01 | 0·00079 |
| 68:01~44:03~07:01~02:01~03:01 | 0·00079 |
| 68:01~44:06~04:03~03:01~03:02 | 0·00088 |
| 68:01~51:01~01:01~01:02~05:01 | 0·00088 |
| 68:01~51:01~08:02~01:01~05:03 | 0·00079 |
| 68:01~51:01~10:01~01:01~05:01 | 0·00088 |
| 68:01~51:01~11:01~05:01~03:01 | 0·0017 |
| 68:01~51:01~15:01~01:02~06:02 | 0·00088 |
| 68:01~51:02~07:01~02:01~02:01 | 0·00079 |
| 68:01~52:01~04:04~03:01~03:02 | 0·00079 |
| 68:01~52:01~07:01~02:01~02:01 | 0·0059 |
| 68:01~52:01~12:02~06:01~03:01 | 0·00088 |
| 68:01~52:01~15:02~01:03~06:01 | 0·0026 |
| 68:01~55:01~07:01~01:01~05:03 | 0·00079 |
| 68:01~55:01~10:01~01:01~05:01 | 0·00088 |
| 68:09~07:02~07:01~02:01~02:01 | 0·00079 |
| 74:01~13:01~04:05~03:01~04:02 | 0·00079 |
| 74:01~15:02~12:02~06:01~03:01 | 0·00088 |
| 74:01~27:07~14:04~01:01~05:03 | 0·00079 |
| 74:01~38:02~15:01~01:02~05:02 | 0·00079 |
| 74:01~38:02~15:04~01:02~05:02 | 0·0017 |
| 74:01~45:01~10:01~01:01~05:01 | 0·00088 |

**Supplementary Table 5: HLA class I/II allele associations with overall diarrhoea**

| **Pathogen** | **Allele** | **Controls** | **Cases** | **Frequency (controls)** | **Frequency (cases)** | **Frequency** | **P-value_Chi-square value** | **Chi-square value** | **Degrees of freedom** | **Odds-Ratio** | **Confidence Interval Lower 95%** | **Confidence Interval Upper 95%** | **P-value** | **P-value_adjusted*** |
| --- | --- | --- | --- | --- | --- | --- | --- | --- | --- | --- | --- | --- | --- | --- |
| Overall Diarrhoea | *B*35:01* | 2 | 58 | 0·014 | 0·055 | 0·034 | 0·17 | 13 | 9 | 4·1 | 1·1 | 35 | 0·034 | 0·31 |
| Overall Diarrhoea | *DRB1*15:01* | 6 | 103 | 0·042 | 0·097 | 0·070 | 0·074 | 13 | 7 | 2·5 | 1·1 | 7·1 | 0·029 | 0·20 |
| Overall Diarrhoea | *DQB1*06:01* | 18 | 88 | 0·10 | 0·18 | 0·14 | 0·23 | 11 | 8 | 1·9 | 1·1 | 3·5 | 0·026 | 0·20 |

*P_adj = Adjusted p-value for multiple comparisons by correcting for number of comparisons for overall diarrhoea.
