# Supplementary material for: HLA class I and II associations with common enteric pathogens in the first year of life
Source: eBioMedicine. 2021 Apr 25;67:103346. doi: 10.1016/j.ebiom.2021.103346 (PMC8093888; doi:10.1016/j.ebiom.2021.103346)
Supplement: Supplementary file 2 [file mmc2.zip › caption for supplementary material.docx]

Supplementary Figure 1: Venn diagram overlap of infants with FDR-significant HLA class I and II alleles: Five HLA loci (A~B~DRB1~DQA1~DQB1) were analysed for associations with 12 common enteric pathogens in 601 infants during the first year of life. Allele-enteric pathogen associations were corrected for multiple comparisons at an FDR-cut-off of 0·15. Numeric values for infants with at least one copy of an allele is shown. The bar graph displays the total number of infants with a corresponding FDR-significant allele. Red = increased susceptibility to infection, blue = decreased susceptibility to infection. Bolded values indicate infants sharing two or more designated significant HLA alleles.
