# Supplementary figures and images for: HLA class I and II associations with common enteric pathogens in the first year of life
Source: eBioMedicine. 2021 Apr 25;67:103346. doi: 10.1016/j.ebiom.2021.103346 (PMC8093888; doi:10.1016/j.ebiom.2021.103346)

Supplementary Figure 1

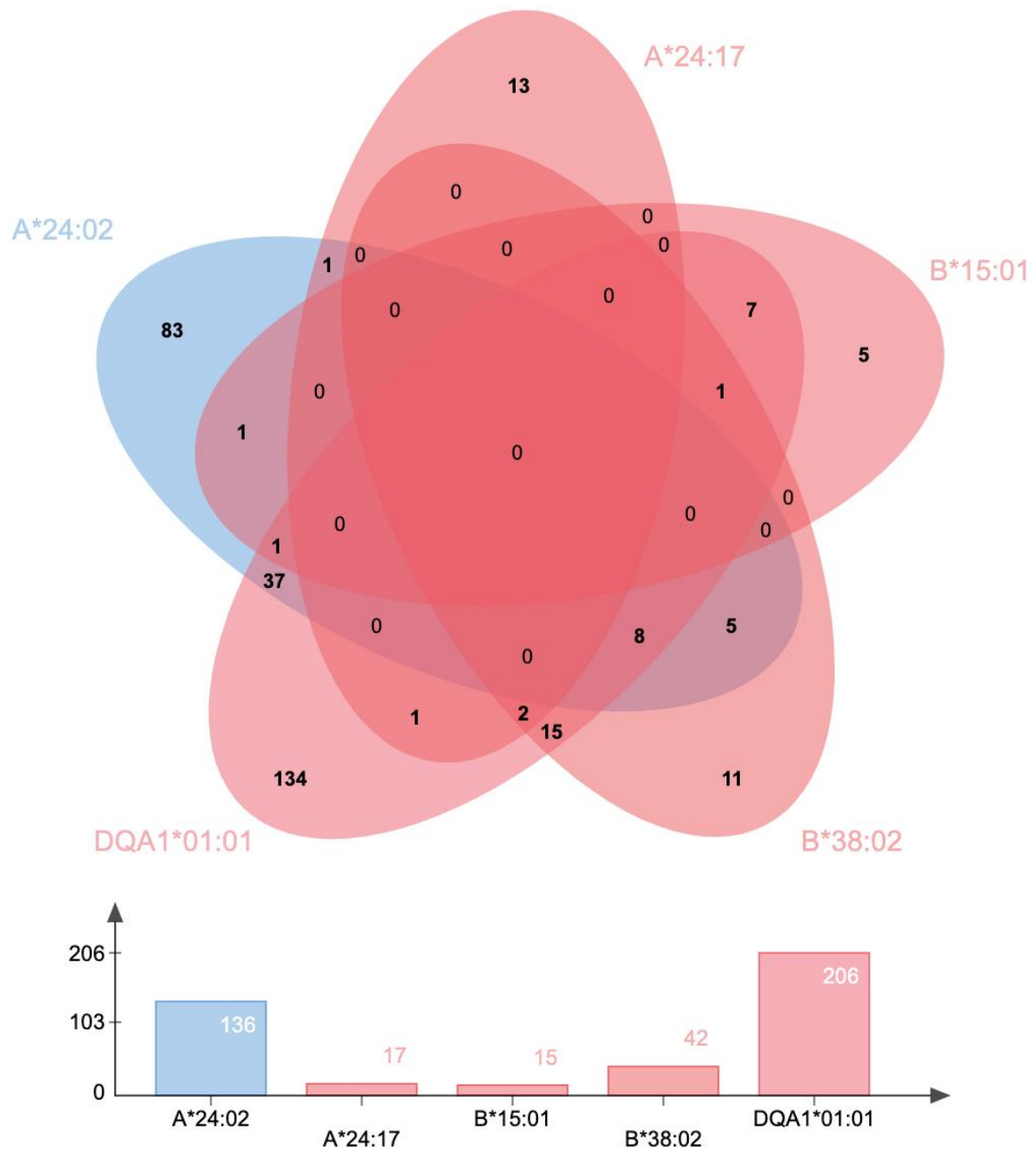

Supplement: Supplementary file 2 [file mmc2.zip › Sup Fig 1.pdf]
